# Pathophysiology and therapy of systemic vasculitides

**DOI:** 10.17179/excli2020-1512

**Published:** 2020-06-18

**Authors:** Massimo Ralli, Flaminia Campo, Diletta Angeletti, Antonio Minni, Marco Artico, Antonio Greco, Antonella Polimeni, Marco de Vincentiis

**Affiliations:** 1 Department of Sense Organs, Sapienza University of Rome, Italy; 2 Department of Oral and Maxillofacial Sciences, Sapienza University of Rome, Italy

**Keywords:** Systemic vasculitides, Kawasaki disease, Takayasu arteritis, Polyarteritis nodosa, Giant cell arteritis, Behcet's disease

## Abstract

Systemic vasculitides represent uncommon conditions characterized by the inflammation of blood vessels that can lead to different complex disorders limited to one organ or potentially involving multiple organs and systems. Systemic vasculitides are classified according to the diameter of the vessel that they mainly affect (small, medium, large, or variable). The pathogenetic mechanisms of systemic vasculitides are still partly unknown, as well as their genetic basis. For most of the primary systemic vasculitides, a single gold standard test is not available, and diagnosis is often made after having ruled out other mimicking conditions. Current research has focused on new management protocol and therapeutic strategies aimed at improving long-term patient outcomes and avoiding progression to multiorgan failure with irreversible damage. In this narrative review, authors describe different forms of systemic vasculitides through a review of the literature, with the aim of highlighting the current knowledge and recent findings on etiopathogenesis, diagnosis and therapy.

## Introduction

Systemic vasculitides represent uncommon diseases characterized by the inflammation of blood vessels that can lead to different complex disorders limited to one organ or potentially involving multiple organs and systems. The annual incidence of vasculitides is 40 to 60 cases per 1 million persons (Reinhold-Keller et al., 2005[196]).

The pathogenetic mechanisms of systemic vasculitides are still partly unknown, as well as their genetic basis. Genome-wide association studies (GWAS) represent the main tests for the identification of the genetic alteration in vasculitides, and have demonstrated a genetic component in many vasculitides such as Takayasu arteritis (Renauer et al., 2015[198]), Kawasaki disease (Kim et al., 2017[125]) and antineutrophil cytoplasmic antibody (ANCA)-associated vasculitides (AAV) (Lyons et al., 2019[153]). However, despite new advances in the comprehension of the genetic basis of vasculitides, their pathogenesis is still partly unknown. 

**Figure 1:**
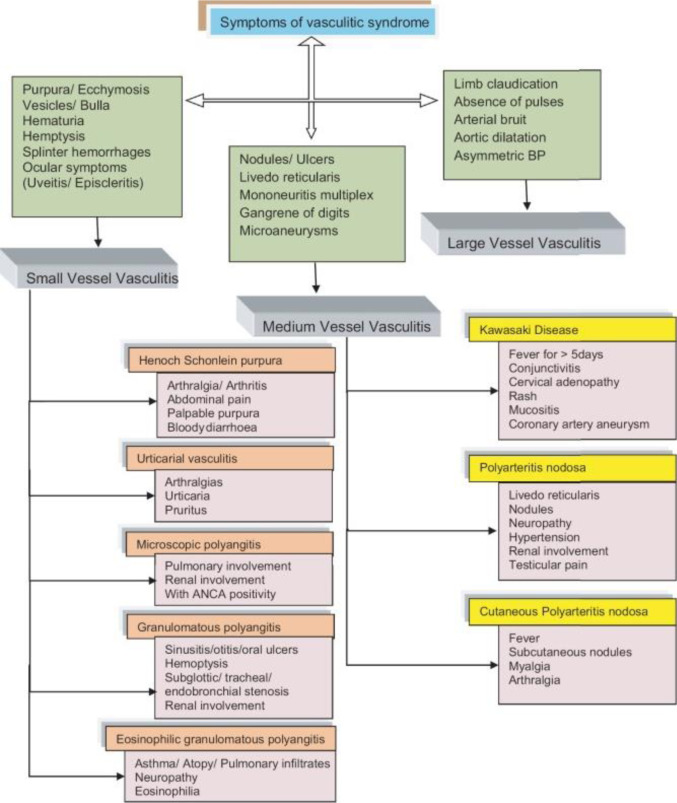
Algorithm for syndromic approach of symptoms in vasculitis (from Sangolli and Lakshmi, 2019; available from: http://www.idoj.in/text.asp?2019/10/6/617/270204)

To date, for most of the primary systemic vasculitides there is not a single gold standard test and no specific diagnostic criteria (Sag et al., 2017[205]). Diagnosis is often made after having ruled out other mimicking conditions, as vasculitides may have specific and non-specific inflammatory symptoms that need to be associated to reach a final diagnosis (Sangolli and Lakshmi, 2019[210]) (Figure 1(Fig. 1)). Current research has focused on new management protocol and therapeutic strategies aimed at improving long-term patient outcomes. 

In this manuscript, authors describe systemic vasculitides through a review of the literature, with the aim of highlighting the current knowledge and recent findings on etiopathogenesis, diagnosis and therapy. After a brief introduction on general classification and epidemiology of systemic vasculitides, the authors describe in details current knowledge on epidemiology, etiopathogenesis, clinical features, diagnosis and treatment of each disease classified following the Chapel Hill Consensus Conference (CHCC) classification (Jennette et al., 2013[108]).

## Classification

**Figure 2:**
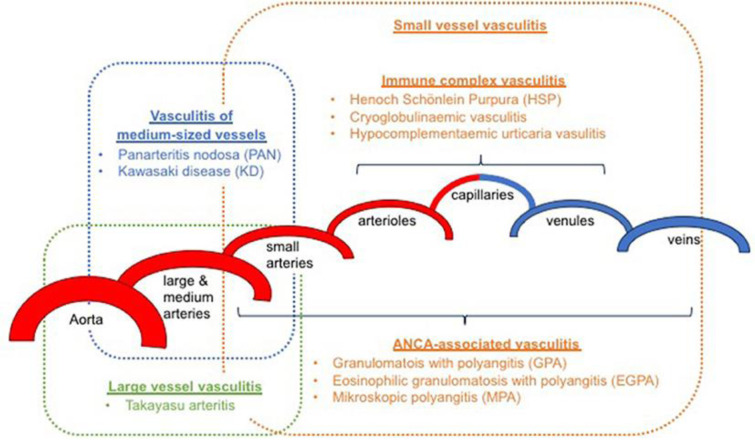
Classification of primary vasculitis based on revised Chapel Hill criteria (2012) and the European League Against Rheumatism (EULAR) / Paediatric Rheumatology European Society (PRES) classification (from Schnabel and Hedrich, 2018; available from: https://doi.org/10.3389/fped.2018.00421)

Systemic vasculitides are defined as systemic diseases of the blood vessels, categorized by the diameter of the vessel that they mainly affect (small, medium, large, or variable) (Schnabel and Hedrich, 2018[216]) (Figure 2(Fig. 2)).

The CHCC in 1994 produced the basis for classification of the above-mentioned disorders (Jennette et al., 1994[107]). After 20 years, the CHCC congregated again to delineate a new nomenclature system that included further classes of vasculitides and improved definitions based on contemporary developments in usage and on the most recent improvements in the comprehension of disease clinical and laboratory signs and mechanisms (Jennette et al., 2013[108]) (Table 1(Tab. 1)).

Several modifications were presented in this new nomenclature paper: small vessel vasculitides were divided into two pathogenetic categories; descriptive names were replaced with eponyms; and new classes were introduced such as variable vessel vasculitis, vasculitis associated systemic disease, single organ vasculitis and vasculitis associated with probable etiology. 

In describing vessel dimensions, the expression “small vessel” indicates the arterioles, capillaries, venules and some veins, “medium vessel” the main visceral arteries and veins and their initial branches and “large vessel” the aorta and its major branches. Nevertheless, some intersection might happen, and arteries of different sizes can possibly be included in more than one category (Jennette et al., 2013[108]). In addition to the multi-organ systemic vasculitides, other types have been described, including single organ vasculitides, vasculitides associated with specific underlying causes and vasculitides associated with systemic diseases. 

## Epidemiology

Finding precise epidemiological data for systemic vasculitides is imperative for numerous reasons. The understanding of pattern of disease is fundamental to plan health services, and the evidence of epidemics or seasonality could be important to understand the pathogenesis and the role of both infectious and environmental factors. Furthermore, the comparison of incidence of vasculitides between different populations can help understanding their genetic risk factors (Watts and Robson, 2018[250]).

Epidemiology of vasculitides has been studied through the identification of a cohort of patients from a precise topographical region based on single referral centers or through the use of databanks. However, the accumulation of data about an adequately large number of patients for uncommon diseases such as vasculitides either needs a long period or a large population, thus making it complex to perform a good-quality epidemiology research for these conditions (Watts and Robson, 2018[250]). 

With rising attention to the incidence of vasculitides in singular groups, precise analysis and identification of ancestry is of utmost importance. The perfect model for the identification of ancestry is the determination of the birthplace for all four of a person's grandparents, but this is frequently not feasible, leading to a reliance on self-reported ancestry. 

These factors have significantly affected the reliability of epidemiological studies in systemic vasculitides and should be taken into account when analyzing epidemiological data on these diseases.

## Large Vessel Vasculitis (LVV)

### Takayasu arteritis

Takayasu arteritis (TA) is systemic inflammatory condition that affects large arteries such as the aorta, its major branches and the pulmonary arteries (Seyahi, 2017[218]; Di Santo et al., 2018[42]). TA is primarily a disorder affecting young adult women, mainly appearing in the second and third decades of life (Tombetti and Mason, 2019[235]).

#### Epidemiology

TA is an uncommon large-vessel vasculitis initially described in Japan and considered for a long time most frequent in population of Asian ancestry. Up to the present time, the highest prevalence described is for Japan (40 per million) (Toshihiko, 1996[236]), though a research in Norway from a multi-ethnic population found a higher prevalence in small cohorts of subjects of African (108 per million) and of Asian (71 per million) ancestry (Gudbrandsson et al., 2017[81]). The incidence in European regions is nearly 1-2 per million per year. 

#### Etiopathogenesis

Inflammatory lesions in TA thicken arterial walls and result in a remodeling of the arterial lumen. Arterial stenoses is more frequent that aneurysmal disease, respectively 90 % and 25 % (Tombetti and Mason, 2019[235]).

TA is a disease with an important morbidity. Mortality is nearly 5 % at 10 years and as high as 27 % in the more severe forms (Ishikawa and Maetani, 1994[103]), while regular activities are compromised in 74 % of patients (Mason, 2010[160]). 

Studies on genetic alterations in TA hypothesized a role for the innate and the adaptive immune systems (Terao, 2016[230]; Renauer and Sawalha, 2017[197]). Both Classes I and II Human Leukocyte Antigen (HLA) loci have been associated with TA (most notably the *HLA-B* locus and the *HLA-B52* allele) (Demir et al., 2019[40]). In Japanese patients, HLA-B*52:01 allele has been confirmed to be related to TA, then confirmed in other populations such as Chinese, Korean, Turkish, European and American (Renauer and Sawalha, 2017[197]).

Through GWAS studies, many susceptibility loci have been recognized such as *RPS9/LILRB3*, *LILA3*, *IL38, *IL12B (Arnaud et al., 2011[9]). Terao et al. in GWAS on 633 TA patients and 5928 controls found a quantity of unreported loci, mainly non-HLA genes (PTK2B, LILRA3/LILRB2, DUSP22, KLHL33) (Terao et al., 2018[231]). Soto et al. found a new link of PTPN22 single-nucleotide polymorphism (R620W) related to susceptibility for TA in a study including 111 patients (Soto et al., 2019[226]). Goel et al. have proven elevated IL6 levels in the vascular lesions and the serum of TA patients (Goel et al., 2017[68]). These findings have reinforced the use of anti-IL6 in TA and helped developing targeted therapies in TA patients. 

Therapy protocols of TA depend on the disease activity. Some cases present in a mild form while others have important morbidity. The main aim of treatment is to limit the inflammatory process and hypertension. Myocardial infarction and stroke have an elevate prevalence in TA and occasionally are the first sign. A meta-analysis described a prevalence of stroke and myocardial infarction respectively of 8.9 % and 3.4 % (Kim and Barra, 2018[124]). TA represented about 10 % of cases of acute ischemic heart disease in females aged < 40 years, consequently, the diagnosis should be ruled out in young patients suffering from cardiovascular complications (Cavalli et al., 2018[28]; Jung et al., 2018[110]). 

#### Clinical features

Commonly, three different phases of TA are documented. In the first phase, there are non-specific constitutional inflammatory symptoms. During this phase, patients can refer fever of unknown origin. In the next phase, patients may refer neck pain and, rarely, thoracic and dorsal pain. The last phase is characterized by decreased or absence of pulses and/or differences in arterial blood pressure between upper extremities, arterial bruits, and intermittent extremity claudication (Keser et al., 2018[120]). The complete form of TA may also be divided into two overlapping phases. While the acute phase represents systemic and initial vascular inflammation, the occlusive phase, which occurs weeks to years later, is characterized by ischemic symptoms (Park et al., 2005[187]; Vanoli et al., 2005[245]). Stroke, transient ischemic attack, and sudden blindness may also be caused by thrombosis of cerebral arteries (Park et al., 2005[187]). Audio-vestibular symptoms have also been reported in cases with TA; they comprise sudden sensorineural hearing loss and vertigo (Kanzaki, 1994[116]; Ralli et al., 2017[193][194]).

It is important to underline that even if involvement of the aorta and its main branches is a typical manifestation of TA, this involvement is not homogeneous in all patients and different types of vessel involvement have been reported (Hata et al., 1996[84]; Keser et al., 2018[120]) (Table 2(Tab. 2)). 

#### Diagnosis

At onset, TA does not present specific signs, with manifestations related to systemic symptoms that may precede the onset of clinically evident signs (Kim and Beckman, 2018[123]). Unfortunately, as in many other vasculitides, there are no gold standard diagnostic tests. The more extensively accepted criteria are those suggested by Ishikawa (1988[102]), and the American College of Rheumatology (ACR) classification criteria (Arend et al., 1990[8]) (Table 3(Tab. 3)). The Ishikawa criteria comprise a mandatory criterion of ≤ 40 years of age at time of diagnosis or beginning of characteristic signs and symptoms (Ishikawa, 1988[102]). Conversely, the ACR criteria were established to differentiate TA from other vasculitides mainly for research purposes, rather than as clinical diagnostic criteria (Arend et al., 1990[8]). 

TA must be distinguished from giant cell arteritis (GCA), an additional relevant cause of inflammatory aortitis. Even if TA and GCA involve common arteries, clinical signs may be different as patients with GCA have superior incidence of ophthalmologic symptoms and jaw claudication. Furthermore, the age of onset is the main different feature between TA and GCA as the former affects an older cohort with mean age at diagnosis of 75 years (Kim and Beckman, 2018[123]).

#### Treatment

Steroids are the main therapy for TA. Immune suppressive agents like cyclophosphamide, azathioprine or biologics should be used for induction of remission, and maintenance treatment may be continued with lower dose steroids and methotrexate (Langford et al., 2017[133]; Nakaoka et al., 2018[175]). With recent studies on pathogenesis and genetic alteration, biologic therapies are increasingly used in TA. Anti-tumor necrosis factor (Anti-TNF) has been described as a successful therapy in TA. Hoffman et al. demonstrated that adding anti-TNF treatment caused an improvement in 14/15 patients (93.3 %) and sustained remission in 10/15 patients (66.7 %) (Hoffman et al., 2004[94]). Youngstein et al. reported that treatment with a TNF-α antagonist, an interleukin (IL)-6 receptor antagonist, or both may be valid in patients with refractory TA (Youngstein et al., 2014[259]). Auspicious results with anti-IL-6 (tocilizumab) and anti-IL-12/23p40 have also been described in recent studies (Mekinian et al., 2018[164]). The scientific community affirmed that IL-6 is the main factor in the inflammatory process of large-vessel vasculitis, and case series have revealed that the humanized monoclonal antibody tocilizumab, which blocks the soluble IL-6 receptor, can produce clinical responses and have a steroid-sparing effect in cases with refractory TA, including patients refractory to anti-TNF (Nakaoka et al., 2018[175]). On the other hand, Terao et al. have demonstrated that Ustekinumab, a monoclonal antibody against IL-12/23p40, may be an effective treatment approach for TA patients, which is supported by genetic association findings (Terao et al., 2016[232]). 

The long-term effects of TA on large vessels may require surgical treatment. Surgery should be considered only during the inactive periods of the disease. Bypass graft surgery, although more invasive, offers enhanced duration of arterial patency (Mason, 2015[159]). Percutaneous balloon angioplasty can be considered a valid treatment approach mainly for recent-onset lesions, while conventional stents appear to be associated with high failure rates (Mason, 2018[158]). However, the use of combined immunosuppression and biologic therapy for refractory TA may decrease requirements for surgical intervention.

### Giant cell arteritis 

Giant cell arteritis (GCA) is a systemic autoimmune condition characterized by granulomatous inflammation of the large and medium arteries (Younger, 2019[258]).

GCA was first described in 1890 by Hutchinson, who defined it as a burning inflamed temporal arteritis that avoided a patient from wearing his hat. Later, Horton and colleagues described GCA as a distinct disease (Horton et al., 1932[95]). In the Western world, GCA is the most frequent primary systemic vasculitis in patients over 50 years of age. GCA was categorized as a large-vessel vasculitis by the 2012 Revised CHCC classification (Jennette et al., 2013[108]).

#### Epidemiology

The main incidence of GCA is in Northern Europe, particularly in the Scandinavian regions and in patients of Scandinavian ancestry (Brekke et al., 2017[19]). Mean age is 70 years, and most affected individuals are over 60 years of age. Women are more affected than men. Incidence of GCA in population aged greater than 50 years is 27 cases in 100,000, although this number will most probably increase in the near future following population aging (Niederkohr and Levin, 2005[178]). The Scandinavian report showed a growth in GCA incidence from 1972 to 1992, but no additional growth up to 2012 (Brekke et al., 2017[19]). GCA is a rare condition in non-European ancestry populations (Kobayashi et al., 2003[130]). 

#### Etiopathogenesis

Environmental and infection agents have been hypothesized to have a role in the etiopathogenesis of GCA (Watts and Robson, 2018[250]). 

Genetic predisposition also plays a central role in GCA. The main genetic risk factor is HLA-DRB1*04 (Carmona et al., 2015[26]); genetic studies have also demonstrated that P4HA2, PTPN22 and PLG were recognized as GCA risk genetic factor (Carmona et al., 2017[27]). It has been recently hypothesized that the expansion of GCA may represent a breach of the immune privilege of the aorta as a result of checkpoint self-consciousness failure (Zhang et al., 2017[261]). 

The first changes in GCA are altered maturation, activation and preservation of antigen-presenting adventitial dendritic cells. These cells sample the contiguous environment for bacterial and viral pathogens within the action of toll-like receptors (Agard et al., 2008[1]; Salvarani et al., 2008[209]).

The typical histologic alteration of GCA comprise elastic lamina fragmentation, intimal thickening and arterial wall inflammation. GCA has been named after the occurrence of multinucleated giant cells, that have been reported in approximately one-half of positive temporal artery biopsies (TABs), in addiction of a granulomatous inflammatory infiltrate composed of CD41 T-cells and macrophages situated at the intima-media junction (Younger, 2019[258]). Other TAB samples show panarteritis associated to lympho-mononuclear cells with sporadic neutrophils and eosinophils without giant cells. 

Typically, arterial wall thickening may provoke incomplete or total occlusion of vessels and ischemic problems, such as anterior ischemic optic neuropathy (Agard et al., 2008[1]). Temporal artery arteritis is not only present in GCA, but also in other conditions such as polyarteritis nodosa, antineutrophil cytoplasm antibody related vasculitis, and atypical polymyalgia rheumatica (Fitzcharles and Esdaile, 1990[57]). 

#### Clinical features

In the early phases, GCA usually is not specific, with a range of symptoms linked to the local effects of systemic and vascular inflammation. GCA symptoms comprise scalp tenderness, headache, and jaw claudication. Headache is specifically localized to the temporal region and is the most frequent symptom. Typically, this type of pain differs from previously experienced headaches, and the patient may refer to the symptom as head pain. Jaw claudication is also a common symptom, described as pain with chewing. For this reason, patients reduce their food consumption and thus lose weight and feel tired (Younger, 2019[258]). Rarely, a patient can observe an inflamed artery in the temporal area. 

Sudden vision loss is relatively common in GCA, as nearly 15 % of patients with GCA experience ophthalmologic difficulties (Hayreh et al., 1998[88]). The disease can present weeks before with ophthalmologic symptoms such as temporary loss of vision following incomplete occlusion of the short posterior ciliary arteries or central retinal artery (Evans and Hunder, 2000[49]). Visual loss is usually described as painless, and can be unilateral or bilateral, partial or complete and may be permanent if untreated. For this reason, it is imperative to suppose GCA in the differential diagnosis for any elderly patient presenting with visual symptoms. Additional ischemic symptoms comprise transient ischemic attack and stroke, micro-embolism, or a combination of distal thrombosis and intimal hyperplasia (Hayreh et al., 1998[88]).

The Diagnostic and Classification Criteria in Vasculitis study in 26 countries reported blindness in at least one eye at 6 months in almost 8 % of patients with GCA. Risk factors for blindness comprise stroke (OR 1⁄4 4.47) and peripheral vascular disorder (OR 1⁄4 10.44) (Yates et al., 2017[256]). 

#### Diagnosis

The ACR 1990 criteria for the classification of GCA require the presence of 3 or more of the following criteria: age > 50 years, temporal artery tenderness, new-onset localized headache, erythrocyte sedimentation rate > 50 mm/h, and abnormal TAB. These criteria have a sensitivity of 93.5 % and specificity of 91.2 % to discriminate GCA from other vasculitides (Murchison et al., 2012[172]).

Acute-phase inflammation markers are frequently considerably raised, and a normocytic normochromic anemia and thrombocytosis may be described in addition to the elevation of liver transaminase levels. The combination of positive TAB and elevated C-reactive protein provides, to date, the main sensitivity and specificity for the diagnosis of GCA.

The gold standard for GCA is the TAB sample (Schmidt, 2013[214]; Younger, 2019[258]); actual sensitivity of unilateral TAB is 87 %.

Temporal artery ultrasound tests are cost-effective, non-invasive, fast and safe, and provide an image of the inflamed temporal artery described by edematous wall swelling. 

#### Treatment

Prompt diagnosis and precocious initiation of medical treatment is of utmost importance since there is the possibility of vision loss if management is deferred. 

Patient should start medical treatment with oral prednisone even before the result of TAB. High-dose corticosteroid protocol is mostly successful in avoiding additional visual loss. Rare cases may have visual reduction during the first days of therapy and, contrariwise, a few patients may present a mild visual function progress when treated with high-dose steroids (Liu et al., 1994[147]; Foroozan et al., 2003[58]). Currently, there is no indication that intravenous high dose corticosteroids are better than oral steroids in limiting visual decline (Hayreh and Zimmerman, 2003[89]).

After the diagnosis of GCA with a positive TAB, the high-dose corticosteroid therapy protocol should be tapered gradually over 1 year, and C-reactive protein (CRP) lab values should be continually monitored. Though it is probable that the visual function is unfortunate after suffering central retinal artery occlusion or ischemic optic neuropathy, the non-ocular signs such as jaw claudication, headache and scalp tenderness may resolve or improve with steroids. In cases in which prednisone's side effects outweigh profits, methotrexate can be used (Chacko et al., 2015[29]). A possibility of persistent ischemic optic neuropathy is present in nearly 7 % of cases and requires prompt re-evaluation.

The mortality with GCA is increased compared to the general population. Mohammad et al. analyzed a large sample with biopsy-proven GCA and found that the incidence of GCA might have decreased over time (Mohammad et al., 2015[167]).

## Medium Vessel Vasculitis (MVV)

### Polyarteritis nodosa 

Polyarteritis nodosa (PAN) is a rare vasculitis characterized by necrotizing inflammatory modification of medium and small muscular arteries, especially at vessel bifurcations (Jennette et al., 2013[108]). The 2012 CHCC established the definition of PAN as a necrotizing arteritis not related with anti-neutrophil cytoplasmic antibodies (ANCAs) of small and medium vascular arteries and not associated to glomerulonephritis or vasculitis in arterioles, capillaries, or venules (Jennette et al., 2013[108]).

PAN alterations result in microaneurysm formation, aneurysmal rupture with hemorrhage, thrombosis, and, therefore, organ ischemia or infarction (Lie, 1989[144]). 

PAN was first described in 1852 by Karl Rokitansky from the Universal of Vienna (Tesar et al., 2004[234]). In 1866, the term “periarteritis nodosa” was proposed by Kussmaul and Maier to define the nodules detected in medium arteries (Tesar et al., 2004[234]).

#### Epidemiology

Until the early 1990s, the term PAN was used to indicate different types of vasculitides that are now called ANCA-associated vasculitis (AAV). PAN can be idiopathic or can be connected with an infective etiology. PAN has been related with various infectious diseases, especially Hepatitis B Virus (HBV) and Human Immunodeficiency Virus (HIV). PAN related with HBV has become infrequent after the diffusion of vaccination protocols and selection of blood products (Mahr et al., 2004[154]). Before HBV vaccination, over one-third of patients with PAN were also HBV positive; this number has now decreased to 5 % in developed countries (Mahr et al., 2004[154]). In Europe, the incidence of PAN every year ranges from 0 to 1.6 cases/million people; prevalence is of 31 cases/million (Hernandez-Rodriguez et al., 2014[93]). The condition onset is typically between the fourth and sixth decade of life, and rarely affects children. A 1.5:1 male preponderance has been described (Hernandez-Rodriguez et al., 2014[93]). 

#### Etiopathogenesis

The pathogenesis of idiopathic PAN is still unclear, nevertheless the optimal clinical response to corticosteroid treatment suggests that immunological system may have a central role (De Virgilio et al., 2016[39]). 

PAN was originally described as an immune-complex disorder for the presence of necrotizing arteritis in *in vivo* models of immune-complex-mediated injury (Yates et al., 2016[257]). 

Nevertheless, glomerulonephritis and complement consumption are not related with PAN. The identification of dendritic cells and the presence of CD4+ lymphocytes in vascular inflammatory infiltrates propose that antigen-specific T-cell mediated immune responses may have a central role in the etiopathogenesis (Guillevin et al., 2011[82]).

The altered endothelial function might reveal direct endothelial cell stimulation (Filer et al., 2003[55]). Activated endothelial cells increase the production of cytokines and adhesion molecules. Blood tests in PAN have documented increases in the blood levels of interferon-γ and IL-2 and amplified serum levels of IL-8, an effective activator of neutrophils (Freire Ade et al., 2004[60]). Laboratory tests have also demonstrated modest increases in TNF-α and IL-1β (Hughes and Bridges, 2002[97]). 

Infectious factors, also, have been associated with development of PAN. HBV is the most frequent and well-defining infection risk factor of PAN, followed by hepatitis C virus (HCV), HIV, cytomegalovirus and parvovirus B19 (Bourgarit et al., 2005[18]).

HBV is related with PAN and has a role in the pathogenesis with at least two mechanisms. Primary, virus replication might provoke damage of the vessel wall (Trepo and Guillevin, 2001[241]). Then, the deposition and the *in situ* development of circulating immune complexes are the main reasons for vascular change. These factors stimulate the complement cascade, which activates neutrophils (Trepo and Guillevin, 2001[241]). The immunological process that underlies PAN is usually detected within 6 months after HBV infection. 

#### Clinical features

The clinical manifestations of PAN range from affecting a single organ to systemic failure (Howard et al., 2014[96]). Every tissue could be affected; nevertheless, for unknown motives, PAN does not involve the lungs (Lhote and Guillevin, 1995[142]) .

The obstruction or break-up of inflamed vessels can generate tissue ischemia or hemorrhage in multiple structures. Therefore, PAN usually presents with collection of clinical indicators, including generic symptoms, such as sickness, fever, weight loss, myalgia and arthralgia.

PAN usually involves peripheral nerves and skin. The manifestations on skin include livedoid, purpura, subcutaneous nodules, and necrotic ulcers. Principal neurological symptom is mononeuritis multiplex, which usually manifests with wrist or foot drop. Furthermore, patients can develop hypertension or renal failure. Wunderlich syndrome is a rare but a typical presentation in PAN; it is characterized by a triad of flank mass, acute flank pain, and hypovolemic shock (Katabathina et al., 2011[117]). Gastrointestinal signs comprise abdominal pain, bowel perforation, weight loss, cholecystitis, pancreatitis and appendicitis.

Bilateral and symmetrical hearing loss has frequently been described in patients with PAN. Hearing loss is mainly sensorineural and, in uncommon cases, it has been reported as the first sign of this condition (Ralli et al., 2018[191][192]). 

#### Diagnosis

Currently, there are no specific biomarkers for PAN. Then, the diagnosis needs the addition of clinical signs, angiographic imaging, and biopsy sample.

Laboratory tests can assist to control the organ damage. The most important tests are serum creatinine, liver function studies, muscle enzyme concentrations, HBV and HCV serologies, and urinalysis. Supplementary laboratory testing, as ANCA, antinuclear antibody, C3 and C4 and cryoglobulins, is valuable in differential diagnosis with others vasculitides (Hernandez-Rodriguez et al., 2014[93]).

The ACR has recognized ten criteria for the classification of PAN (Lightfoot et al., 1990[145]). If at least three of the following criteria are present, the sensitivity and specificity for the diagnosis of PAN is between 82 % and 87 %: inexplicable weight loss superior than 4 kg, livedo reticularis, testicular pain or tenderness, myalgias, weakness and tenderness of muscles, mononeuropathy or polyneuropathy, new-onset diastolic blood pressure > 90 mmHg, elevate levels of serum blood urea nitrogen or creatinine, indication of HBV infection via serum antibody or antigen serology, typical arteriographic irregularities not causing from non-inflammatory disorder processes, vessel biopsy comprehending polymorphonuclear cells.

The evidence of focal, necrotizing inflammation of medium or small arteries, especially in bifurcations points, is considered the gold standard for the diagnosis of PAN. Skin is the preferred tissue for the biopsy diagnosis. Usually, for the diagnosis, arteriography and cross-sectional imaging of the mesenteric or renal circulation can be used instead of tissue biopsy (Balow, 1985[14]). Arteriography can help in the diagnosis, demonstrating irregular constrictions and multiple aneurysms in the larger vessels with occlusion of smaller penetrating arteries. Additional findings include multiple 1-5 mm peripheral aneurysms, occlusions, irregular stenoses, and/or diffuse wall thickening of medium-sized arteries.

#### Treatment

Glucocorticoids are the main therapy for PAN with remission in 50 % of cases. With the supplement of cyclophosphamides, remission approaches 90 % of cases (Howard et al., 2014[96]). HBV-PAN necessitates the supplement of antivirals. In a rare cases, anti-TNF agents have been considered as a therapeutic support for PAN; though, their indication is not clear (Keystone, 2004[122]; Pagnoux et al., 2010[186]). 

### Kawasaki disease

Kawasaki disease (KD) is a systemic vascular disease that affects typically medium and small vessels (Ozen et al., 2006[185]; Dietz et al., 2017[44]). KD is typically self-limited and the highest incidence is seen in children under 5 years of age (Nakamura, 2018[173]). 

KD is currently recognized to be a systemic vasculitis with a particular preference for the coronary arteries and, in Western countries, is the most frequent reason of heart disease in children. The etiopathogenesis of KD is not completely clear despite recent research has focused on causes and treatment strategies (Greco et al., 2015[75]).

#### Epidemiology

KD was first defined by Kawasaki (Kawasaki, 1967[118]) in Japan in the 1960s but afterwards it has been documented in several geographical areas; there are, nevertheless, distinct manifestations between different regions. Inhabitants of Asian ancestry have the maximum incidence of KD. Makino et al. found that the incidence of KD keeps growing in Japan; in this country incidence rate was 243.1 per 100,000 population aged 0-4 years in 2011, increased to 264.8 in 2012 (Makino et al., 2015[155]). Cumulative incidence of KD in Japan is 1.5/100 boys and 1.2/100 girls of age 10 years (Nakamura et al., 2018[173]). In contrast to other rheumatic disorders, there is evidence from different studies that KD affects more boys than girls.

In North-East Asian countries, such as Korea and Japan, the described incidence of KD is much higher than that in Western countries with a growing trend, while in Australia, USA and Europe the earlier detected growth in the incidence of KD seems to have plateaued. 

#### Etiopathogenesis

Although an association between KD and environmental and genetic factors has been demonstrated, the exact etiology of this condition is still unclear (Greco et al., 2015[75]; Elakabawi et al., 2020[45]). The seasonal cluster is evident among different ethnical groups, such as summer/spring peak in China and winter/spring peak in Japan (Burns et al., 2013[23]). The seasonal variation may be determined by different infectious agents (Burns et al., 2013[23]; Rypdal et al., 2018[203]). Recent studies have speculated that tropospheric wind patterns and air pollution may trigger the immunopathological pathways in genetically susceptible children by variable agents (McCrindle et al., 2017[162]). In addition, Severe Acute Respiratory Syndrome - Coronavirus 2 (SARS-CoV-2) infection has been reported to trigger KD although evidence is still sparse (Licciardi et al., 2020[143]; Sardu et al., 2020[211]).

The observation of an increased incidence among Japanese descent residing outside of Japan and the improved incidence of a history of KD between the parents of a KD patient suggest a genetic component in KD (Uehara et al., 2004[243]). 

GWAS studies have tried to explain the molecular alteration in KD. Farh et al. proposed the role of B‐linage cells in KD pathogenesis (Farh et al., 2015[51]). The decreased expression of messenger RNA from the common risk haplotype of *BLK* in B cells (Simpfendorfer et al., 2015[224]) and the increased expression of full‐length, membrane bound isoform of CD40 (Field et al., 2015[53]) on B‐cells can provoke enhance of B cell activity (Onouchi, 2018[181]).

#### Clinical features

Typically, patients with KD have fever ranging from 38 °C to 40 °C and frequently with no prodromal symptoms such as rhinorrhoea, cough and sneezing. Bilateral conjunctival injection without exudate appears within 2-4 days from the beginning of the disease. Modifications in oral cavity are typically defined by bleeding of the lips, redness, fissuring and dryness, strawberry-like tongue without vesicles or pseudo-membrane formation, aphthae or ulcerations, and diffused erythema of the oropharyngeal mucosa. Polymorphous erythema develops on the body and/or extremities, from the first to the fifth day after the onset of fever. Different kind of exanthema have been described: a morbilliform maculopapular rash, an urticarial exanthema with big erythematous plaques or, less frequently, an erythema multiforme-like with central clearing or iris lesions. After a week from the onset, diffuse erythema occurs on the palms and soles of hands and feet. Cervical lymphadenopathies have been reported in nearly half of KD patients in the USA and 70 % in Japan, whereas the other principal symptoms have been described in > 90 % of patients (Kawasaki, 2006[119]). Lymphadenopathies are usually unilateral, and of 2-5 cm in diameter.

#### Diagnosis

In patients with KD, the main symptoms for the diagnosis are persistent fever in association with a polymorphous exanthema, modifications of lips and oral cavity, cervical lymphadenopathy, non-purulent conjunctival injection, and alterations in extremities as redness and swelling of the palms and desquamation during the subacute phase (McCrindle et al., 2017[162]). 

According with the American Heart Association (AHA) guidelines, “complete” KD is described as persistent fever ≥ 5 days and at least 4 other symptoms. It is essential to understand that clinical manifestations can happen sequentially or concurrently. These guidelines have emphasized the importance of the coronary arteries imaging (McCrindle et al., 2017[162]). The AHA has produced a process to make the diagnosis of “incomplete” KD if three or less diagnostic criteria are present. 

Specific laboratory tests for the diagnosis of KD are not available, and diagnosis is based only on clinical observation and symptoms. Recent studies have proposed inflammatory, proteomic, and genetic biomarkers that may be helpful in the diagnosis and management of KD. Parthasarathy et al., in their review, analyzed biomarkers that may be utilized to discovery the gold standard test for KD diagnosis. The results suggest that NT-proBNP is currently a very favorable biomarker for future investigation; additional studies are necessary to find specific tests that allow an early and accurate diagnosis of KD (Parthasarathy et al., 2015[188]).

#### Treatment

The gold standard for treatment of KD is a high dose of 2 g/kg intravenous immunoglobulin (IVIG), given over 8-12 h (Newburger et al., 1991[177]). The aim of therapy is inhibition of the progress of coronary artery abnormalities. The effectiveness of IVIG is probably due to the activation of an immature myeloid population of dendritic cells that produces IL-10, the modulation of T regulatory cells, and the decrease of cytokine production (Burns and Franco, 2015[22]). Early treatment with IVIG can considerably reduce the occurrence of coronary artery abnormalities (Terai and Shulman, 1997[229]). In addition to IVIG, high-dose aspirin is recommended by the AHA, though confirmation for further risk reduction for coronary artery aneurysms is lacking (Dietz et al., 2017[44]).

Most of the patients respond rapidly to IVIG. The Kobayashi score (Kobayashi et al., 2006[131]) is the most popular scoring system to predict the IVIG-resistant patients while this score did not prove useful in western ethnicities. According to the AHA guidelines, high-dose pulse steroids, infliximab, cyclosporine, and anakinra should be considered in patients who have failed response to standard therapy (McCrindle et al., 2017[162]). 

Multiple studies have examined the outcomes after KD. Baker et al. studied 110 KD children and reported that general physical and psychosocial health characteristics of KD patients without coronary artery aneurysms were similar to the general population (Baker et al., 2003[13]). Only cases with giant coronary artery abnormalities had a lower physical score. However, parents affirmed lower health perception. King et al. studied 38 KD patients and found deficits in attentional behavior and internalizing, but did not describe significant differences on academic performance and cognitive development (King et al., 2000[126]). Nevertheless, self-report by the older KD children did not demonstrate differences with controls (Tacke et al., 2012[227]; van Oers et al., 2014[244]) .

## Small Vessel Vasculitis (SVV)

### Antineutrophil cytoplasmic antibody (ANCA)-associated vasculitis (AAV) 

Anti-neutrophil cytoplasmic antibody (ANCA)-associated vasculitis (AAV) is a condition characterized by necrotizing inflammation of the small vessels, the rareness of immune deposits and a link with circulating ANCAs. Different classes have been defined: granulomatosis with polyangiitis (GPA - Wegener's granulomatosis), microscopic polyangiitis (MPA), eosinophilic GPA (EGPA - Churg-Strauss syndrome) and single organ disease including renal-limited vasculitis characterized by small-size vessels inflammation and presence of ANCA (Wendt et al., 2013[251]; Iannella et al., 2016[99]). 

#### Epidemiology

In Europe, MPA, GPA and EGPA have annual incidence rates of 2.4-10.1, 2.1-14.4 and 0.5-3.7 per million respectively, while the prevalence of AAV is estimated at to range between 50-180 per million (Watts et al., 2015[249]). The mean age of disease onset is 60 years, and it is slightly more common in men. The 5-year survival rates for MPA, GPA and EGPA are assessed to be 45 %-76 %, 74 %-91 % and 60 %-97 %, respectively (Robson et al., 2015[199]). In children, AAV has a higher incidence of morbidity, relapse and damage when related to adult AAV patients (Lee et al., 2019[137]). Subglottic stenosis, fever, ischemic abdominal pain and nasal cartilage injury are more frequent in paediatric patients while myalgia and peripheral neuropathy are less common (Greco et al., 2015[78]; Ralli et al., 2018[191][192]; Lee et al., 2019[137]). 

In the Caucasian area, the incidence of AAV is comparable, although modern studies in non-European ancestry populations suggest a lower incidence (Gardner-Medwin et al., 2002[65]). A multi-ethnic study from Chapel Hill in patients in the United States showed that GPA is rare in African Americans related to that in white Caucasians (Piram et al., 2017[190]). 

#### Etiopathogenesis

The main pathogenetic factor in AAV is the interaction between triggering environment exposure and genetic predisposition. Antibodies targeting neutrophil Proteinase-3 (PR3) and Myeloperoxidase (MPO) are common in AAV. PR3-ANCA is highly sensitive for GPA being present in 80-95 % (Greco et al., 2016[77]; Chen et al., 2018[30]; Dick et al., 2018[43]; Heeringa et al., 2018[90]; Tracy et al., 2019[238]). MPO-ANCA positivity has been noticed in 40 % of EGPA cases. In addition, the presence of MPO-ANCA has been described in up to 70 % of MPA patients. However, there are ANCA negative cases. To date, three promising biomarkers have been identified for distinguishing AAV and non-AAV patients: CXCL13, matrix-metalloproteinase-3 and tissue-inhibitor of metalloproteinase-1 (Monach, 2014[168]). 

Lilliebladh et al. (2018[146]) have examined the incidence of several CD4+ T cell subsets in addition to chemokines and effector cytokines in plasma from AAV cases, with active disease and in remission, concerning healthy blood donors and patients with a kidney transplant due to a non-inflammatory disease. AAV patients had minor percentages of naive CD4+ T cells and an increase of effector memory CD4+ T cells when relating to healthy blood donors but no differences were discovered between patients with a kidney transplant and AVV cases.

Infectious and environmental factors have been related to AAV (Lazarus et al., 2016[136]). It has been detected that GPA was the main type in the UK, while MPA was the main type in Japan (Kobayashi and Fujimoto, 2013[129]). Chronic work-related contact to silica is related to an amplified possibility of developing AAV. It has been hypothesized that this occurs because silica induces a significant inflammatory response that stimulates neutrophil migration and concurrent development of antibodies against neutrophil constituents. Nasal carriage of *Staphylococcus aureus* is becoming progressively accepted as a possible “second hit” essential to break tolerance and produces relapse in PR3-AAV patients (Laudien et al., 2010[135]; Salmela et al., 2017[208]). Molecular mimicry between PR3 and parts of *Staphylococcus aureus* clarify the association. Furthermore, the decrease of relapse rate with antibiotic prophylaxis confirms these findings. The linkage between infections and pathogenesis has also been supported by the finding of Lysosome-Associated Membrane Protein 2 (LAMP-2) autoantibodies, which is similar to bacterial adhesion film (Kain et al., 2008[113]). 

GWAS studies have confirmed the role of ANCA in AAV pathogenesis. Lyons et al. (2019[153]) have proven that anti-PR3 ANCA was related with HLA-DP, the genes encoding alfa-1-antitrypsin (SERPINA1) and PR3 (PRTN3), whereas anti-MPO ANCA was related with HLA-DQ. This study has therefore confirmed the genetic distinctions between GPA and MPA regarding ANCA serotype (Lyons et al., 2019[153]). 

#### Clinical features

The onset of disease is usually characterized by rhinitis, sinusitis, malaise and arthralgia. Prodromes frequently precede pulmonary-renal syndrome by weeks or months. 

AAV have been named pulmonary-renal syndromes because they cause pulmonary hemorrhage and hematuria. Furthermore, otitis media and sinus disorder in adults are common features of GPA (Csernok et al., 2006[34]; Kallenberg, 2007[114]). EGPA usually presents with dermatologic manifestations, peripheral eosinophilia and asthma. There are numerous types of unusual presentations of AAV. For GPA, cases of cardiac arrhythmias and myocarditis have been described (Kallenberg, 2007[114]). In the literature, cases of gastrointestinal disease associated with AAV, including AAV imitating inflammatory bowel disease and surgical abdominal disease have been reported (Csernok et al., 2006[34]). Pulmonary hemorrhage and acute kidney injury are indicators of severe vasculitis that has a high risk for morbidity and mortality (Lamprecht et al., 2018[132]). In case of rapidly progressive glomerulonephritis, it is necessary to perform a biopsy and initiate high-dose cytotoxic therapy (Lamprecht et al., 2018[132]). The higher level of anti-PR3 is another risk factor for unfortunate outcome (Westman et al., 2003[252]).

#### Diagnosis 

The absence of defined criteria for the diagnosis of AAV leads to a substantial diagnostic deferral of more than 6 months in a third of cases.

AAV can be typically supposed in cases with characteristic clinical manifestations such as fever, kidney alteration, laboratory inflammation markers and disease of upper and lower respiratory tract (Jennette and Falk, 1997[106]). 

GPA and MPA have overlying symptoms although differences are also present. Patients with GPA often manifest extravascular granulomatous lesions that are not seen in MPA. In addition, the involvement of ear, nose and throat has been more frequently reported in GPA compared to MPA (Jennette and Falk, 1997[106]).

MPO-ANCA are more frequent in MPA and PR3-ANCA are typically present in GPA patients. In cases of GPA that include ear, nose and throat symptoms, it may be challenging to discriminate GPA and MPA. In these patients, frequently imaging tests demonstrated the occurrence of pseudotumor with demolition of nasal sinus and orbital walls. In the presence of an isolated orbital or sinus mass, the diagnosis of GPA might be proven with biopsy or after surgery. ANCA could be identified with immunofluorescence method or ELISA (Schmitt and van der Woude, 2004[215]); however, these autoantibodies can be negative throughout immunosuppressive treatment. Biopsy of kidney, nose and lung may be used to make the diagnosis, but histological samples are not essential in all cases.

#### Treatment

Treatment of AAV is organized into two phases: induction and maintenance (Iannella et al., 2016[99]). The European League Against Rheumatism (EULAR) recommendations for the treatment of AAV have been published in 2016 (Yates et al., 2016[257]). In cases with non-organ threating AAV, association of glucocorticoids and either methotrexate or mycophenolate mofetil should be preferred for remission-induction (Yates et al., 2016[257]). In patients that develop severe organ complication, treatment with a combination of glucocorticoids and either cyclophosphamide or rituximab is suggested for the remission induction (Yates et al., 2016[257]).

A combination of low-dose glucocorticoids and either methotrexate, azathioprine, rituximab, or mycophenolate mofetil is necessary for the remission maintenance management. Considering glucocorticoids sparing strategies, Miloslavsky et al. (2018[166]) proposed a pilot trial (the SCOUT trial) with 20 patients affected by GPA or MPA. That received a remission induction treatment with rituximab 375 mg/mq weekly for 4 weeks and an 8-week glucocorticoids course. Limitations of the study were the small size of sample and the exclusion of more severe clinical manifestations such as alveolar hemorrhage. Despite its limitations, the SCOUT pilot trial demonstrated that reducing glucocorticoids dosage in AAV patients during remission induction is not only possible but can effectively reduce treatment-related damage and side effects. Some auspicious results with targeted therapies have been described in EGPA cases. Recently, improved remission rates with mepolizumab (a monoclonal antibody against IL-5) have been described in EGPA patients (Faverio et al., 2018[52]). Jachiet et al. reported that Omalizumab may have a corticosteroid-sparing effect in EGPA patients with sinonasal and/or asthmatic manifestations (Jachiet et al., 2016[104]). 

#### Anti-glomerular basement membrane disease

Anti-glomerular basement membrane (anti-GBM) vasculitis is a small vessel disease that involves glomerular capillaries, thus producing quickly advanced renal failure, and pulmonary capillaries causing lung hemorrhage, or both. The identification of circulating and deposited antibodies against basement membrane antigens is a typical finding in anti-GMB (McAdoo and Pusey, 2017[161]). “Goodpasture disease” is also used to define this disorder, as it has been first described in 1919 by Ernest Goodpasture (Henderson, 2009[92]). 

#### Epidemiology

The incidence of anti-GBM disease is still unclear; European populations have an incidence of < 1 per million population/year, mainly for single-center biopsy- or serology-based series (McAdoo and Pusey, 2017[161]).

#### Etiopathogenesis

The GBM is a complex of type IV collagen molecules, each made up of triple-helical protomers of α3, α4, and α5 chains. In anti-GBM disease, the focal target of the autoimmune response has been documented as the non-collagenous (NC1) domain of the α3 chain of type IV collagen (α3[IV]NC1) (Saus et al., 1988[213]; Turner et al., 1992[242]). 

The presence of this antigen in glomerular and alveolar capillaries can cause the reno-pulmonary signs (Gulati and McAdoo, 2018[83]). EA and EB have been identified as the two main autoantibody epitopes within the autoantigen (Netzer et al., 1999[176]), which are typically sequestered within the non-collagenous domains of the triple helix of α3, 4, and 5 chains.

T cells have a part in disease etiopathogenesis. T cells may supply cell-mediated glomerular injury, and glomerular T lymphocytes can be found in kidney biopsy samples taken from patients during the acute phase (Bolton et al., 1987[17]). The HLA association and the presence of high-affinity, class-switched autoantibodies, indicate that T cells may contribute in the expansion of the autoimmune response (McAdoo and Pusey, 2017[161]). 

#### Clinical features

Patients typically describe abrupt onset of oliguria or anuria. Hematuria or tea-colored urines are usually observed. Actually, the most frequent symptom (90 %) is rapidly progressive glomerulonephritis. Lung hemorrhage is found in 40 % to 60 % of patients, while a smaller number of cases might present with only lung disorder. Although pulmonary hemorrhage may be minor, it is often severe and life threatening. 

#### Diagnosis

The identification of anti-GBM antibodies is fundamental for the diagnosis; antibodies can be found in serum or deposited in tissue with or without indication of alveolar hemorrhage (McAdoo and Pusey, 2017[161]).

Anti-GBM antibodies are characteristically recognized using enzyme immunoassays or bead-based fluorescence assays that use recombinant or purified human or animal GBM preparations as antigenic substrate (McAdoo and Pusey, 2017[161]). The most sensitive test is Western blotting, while an alternative test is indirect immunofluorescence using normal kidney tissue, though this necessitates extra input from a kidney pathologist, and it may return false negative results. It is important to underline that nearly 10 % of cases do not express circulating antibodies with traditional assays; therefore, serologic methods must not be used as the only test of diagnosis when renal sample is accessible (Gulati and McAdoo, 2018[83]).

The gold standard for identification of anti-GBM disease is direct immunofluorescence for immunoglobulins on frozen kidney biopsy. It is very helpful to identify deposited antibodies (Gulati and McAdoo, 2018[83]), classically showing a strong linear ribbon-like appearance. A relevant caveat is that fluorescence might appear negative in cases presenting with severe glomerular inflammation, where the pattern is so unsettled that the architecture might not be renowned (McAdoo and Pusey, 2017[161]). 

Diffuse alveolar hemorrhage is found clinically and with radiologic imaging. Broncho-alveolar lavage might discover hemosiderin-laden macrophages. However, functional testing as the alveolar carbon monoxide transfer factor might contribute with the differentiation of alveolar hemorrhage from other causes of lung infiltration. 

#### Treatment

Regular management to remove pathogenic autoantibodies comprises plasmapheresis, corticosteroids and cyclophosphamide, to constrain further autoantibody production and stop organ inflammation (Rovin et al., 2019[202]). 

Immunoadsorption might appear better than plasma exchange for the elimination of pathogenic autoantibodies, but it has been used only in small series (Biesenbach et al., 2014[16]; Zhang et al., 2014[262]).

Additional treatments have been proposed. Some studies suggested to use rituximab, mycophenolate mofetil and cyclosporine in addition to classic treatment (Kiykim et al., 2010[128]; Mori et al., 2013[169]; Touzot et al., 2015[237]). Selected cases might require organ support; in bigger series, almost half of cases necessitate hemodialysis at the onset of the disease (Levy et al., 2001[140]). 

Long-term follow-up studies showed improvements in the last decade. Treatments with plasmapheresis, steroids, and immunosuppressive agents have intensely improved prognosis (Shah and Hugghins, 2002[219]). The 5-year survival rate surpasses 80 % and less than 30 % of cases necessitated long-term dialysis (Shah and Hugghins, 2002[219]).

Relapse is uncommon and occurs in < 3 % of patients (Levy et al., 2001[140]). Relapse is commonly connected with hydrocarbons and cigarette smoke (Gu et al., 2016[80]), and prevention of these risk factors are indispensable for the correct management.

### Cryoglobulinemic vasculitis 

Cryoglobulins (CGs) are antibodies that precipitate *in vitro* at temperatures < 37 °C and dissolve after rewarming. Cryoglobulinemic vasculitis (CV) is a form of vasculitis caused by the deposition of CGs in the blood vessels (Desbois et al., 2019[41]).

Brouet's classification divided the disease in three subtypes, on the base of immunoglobulin presentation. Type I cryoglobulinemia includes single monoclonal immunoglobulins, generally immunoglobulin M (IgM), infrequently IgG or IgA, while type II and type III are categorized as mixed cryoglobulinemia since they include two types of immunoglobulins (usually IgG and IgM). Type II mixed cryoglobulinemia contains a pattern of monoclonal and polyclonal immunoglobulins, while type III mixed cryoglobulinemia includes IgM and IgG, both polyclonal (Brouet et al., 1974[20]).

Type I cryoglobulinemia represents almost 15 % of cases of CV and is frequently related to monoclonal gammopathy of undetermined significance (MGUS) and with lymphoproliferative disorders such as myeloma or B-cell lymphoma (Silva et al., 2019[223]). 

Mixed cryoglobulinemia (MC) comprehend both type II and type III cryoglobulinemia. Of all cryoglobulinemias, 50 %-60 % are from type II and 30 %-40 % are type III.

MC is related with different disorders, particularly autoimmune diseases such as Sjögren's syndrome and Lupus Erythematosus, as well as HCV infection or B-cell malignancies (Silva et al., 2019[223]).

About 10 % of cases of MC are considered as idiopathic; this percentage grows to 25 % in HCV-negative patients (Desbois et al., 2019[41]).

#### Epidemiology

CV is uncommon, but its occurrence might be underestimated for the different clinical symptoms that are unspecific for CV. The prevalence has been assessed at nearly 1 in 100,000, seems more frequent in patients aged 45-65 years, with a higher incidence in women (Terrier et al., 2013[233]). 

#### Etiopathogenesis

Type I cryoglobulinemia is characterized by monoclonal CGs produced by lymphoproliferative disease. Contact with cold provokes precipitation that induces vessel obstruction and inflammatory vasculitis. Chronic inflammatory state in MC leads to hyperactivation/hyperproliferation of B-cells, which provoke the proliferation of CGs (Takada et al., 2012[228]). Little is known about the pathophysiology of CV, while the ethology is well described for HCV associated mixed cryoglobulinemia (Roccatello et al., 2018[201]). In the latter, HCV envelope glycoproteins E1 and E2 allow the virus to infect hepatocytes and lymphocytes through the CD81 cell receptor, and chronic HCV infection stimulates intrahepatic and circulating B cells (Pileri et al., 1998[189]). 

#### Clinical features

CV is asymptomatic in many patients, while the number of symptomatic cases ranges between 2 % and 50 % (Trejo et al., 2001[240]). The main symptoms of type I CGs are hyperviscosity and/or thrombosis. Therefore, its most common presentations are the Raynaud's phenomenon, distal gangrene, ischemic ulcers, purpura, livedo reticularis, cold-induced urticaria, headache, retinal hemorrhages and encephalopathy (Ramos-Casals et al., 2000[195]).

Patients with MC may also present nonspecific systemic and musculoskeletal symptoms, like cutaneous vasculitis and neuropathy. Meltzer triad can be found in CV, and includes purpura, weakness and arthralgia, but only occurs in a fraction of patients (Ramos-Casals et al., 2000[195]).

The most frequent symptom is recurrent palpable purpura, occurring in nearly 90 % of cases. It comprises recurrent lesions situated in lower limbs and sometimes spreading to the abdomen. 

Joint manifestations (50 %-75 %) appear as non-migratory pain that includes the hands and knees in a bilateral and symmetric configuration (Desbois et al., 2019[41]). 

Neuropathy appears as paraesthesias or burning pain in legs that might worsen at night. 

Renal symptoms typically develop during or shortly after cutaneous manifestation, and present with varying degrees of microhematuria, hypertension, proteinuria, and/or renal failure (Roccatello et al., 2007[200]). About 20 % of cases develop nephritic syndrome, and occasionally acute renal failure or nephrotic syndrome.

#### Diagnosis

The diagnosis of CV is based on the manifestation of both clinical vasculitis signs and laboratory detection of serum CGs, although there are no standardized diagnostic criteria (Lerner and Watson, 1947[139]). Some laboratories identify CG using immunofixation or immuno-electrophoresis and quantify their level by determining the cryocrit as the total volume percentage. Immunoblotting for immuno-chemical characterization is a sensitive method that allows a full identification in 98 % of cases (Cacoub et al., 2015[24]).

Rheumatoid factor (RF) and complement (C3, C4, CH50) are used for the diagnosis. RF is typically increased in serum of type II MC. Type I cryoglobulinemia characteristically manifests few serological complement alterations; while MC provokes reduction of CH50, C1q, C2 and C4 (Silva et al., 2019[223]). Antinuclear antibodies (ANA) and antineutrophilic cytoplasmatic antibodies (ANCA) should be primarily assessed during initial evaluation (Sargur et al., 2010[212]).

The gold standard test for diagnosing CV is histological evidence of vasculitis. Biopsy is frequently performed in affected tissues such as skin, kidney and nerves. Skin samples typically show leukocytoclastic vasculitis, the hallmark pathological feature, with T and mononuclear infiltrating cells. Endoneurial vasculitis, characterized by vessel wall destruction and axonal degeneration, can be found at neural biopsies. Renal biopsies usually show membranoproliferative glomerulonephritis with subendothelial immunoglobulins and complement deposition (Sargur et al., 2010[212]).

#### Treatment

Treatment focuses on the underlying symptoms and is performed only in symptomatic patients. Treatment options comprise steroids, thalidomide, lenalidomide, bortezomib, or alkylating agents for myeloma patients. In patients with Waldenström's macroglobulinemia, bortezomib is often used as first-line therapy. The treatment management of IgG MGUS is with myeloma drugs that target plasma cells; IgM MGUS is usually treated with rituximab. Plasma exchange treatment is reserved to cases with severe kidney manifestations or extensive leg necrosis (Muchtar et al., 2017[171]; Sidana et al., 2017[221]).

There are not evidence-based treatment recommendations in non-HCV MC. Cases with life-threatening manifestations require immunosuppressive therapy such as high-dose steroids, cyclophosphamide, rituximab and/or plasmapheresis (Muchtar et al., 2017[171]). 

HCV-CV activity often relates with viremia, and management should focus on the causal agent with steroids and antivirals. The selection of antiviral drugs must be done according to current guidelines (Muchtar et al., 2017[171]).

The clinical history of CV is unpredictable and is contingent on concomitant diseases and complications. Death typically occurs after an extended course of vasculitis, lasting years. Watchful monitoring of life-threatening complications should be carried out in all cases with CV.

### Immunoglobulin A vasculitis (IgAV) / Henoch-Schonlein Purpura

Immunoglobulin A vasculitis (IgAV), previously known as Henoch-Schonlein Purpura, is an inflammatory condition that affects small blood vessels, venules, or arterioles with IgA1-dominant immune deposits (Calvino et al., 2001[25]; Lau et al., 2010[134]; Heineke et al., 2017[91]).

#### Epidemiology

IgAV is the most frequent type of childhood vasculitis. The pediatric form is usually benign and self-limited (Gonzalez-Gay and Garcia-Porrua, 2001[69]), while in adults it is uncommon but frequently characterized by rapid clinical progression (Gonzalez-Gay et al., 2018[70]).

The incidence during childhood ranges from 3 to 28 per 100,000/year, mainly between the ages of 4 and 6 years (70 per 100,000) (Gardner-Medwin et al., 2002[65]). Piram et al. in their study registered an annual incidence of 18.6/100,000 children in an area on Paris (Piram et al., 2017[190]). 

IgAV is less common in the adult population with an incidence of approximately 1.4 per million adults (Watts and Robson, 2018[250]). Adult-onset IgAV is more common in males (62 %) and has a mean onset age of 51 years (Audemard-Verger et al., 2017[10]). 

#### Etiopathogenesis

The exact pathophysiology of this disorder is still largely unknown, except that it has been hypothesized that abnormal immunoglobulin A (IgA) plays a central role. Atypical IgA1 glycosylation is assumed to be the principal reason in the etiopathology (Oni and Sampath, 2019[180]), and raised serum galactose-deficient IgA1 levels are detected in IgAV (Kiryluk et al., 2011[127]). It is supposed that there might be anomalies in the fundamental genes in the glycosylation pathway. This altered glycosylation results in the exposure of residues that induce a humoral autoimmune response (Oni and Sampath, 2019[180]). 

GWAS studies dedicated to IgAV have been recently published, but the molecular alterations have not been completely explained. The HLA region is the principal genetic factor related with IgAV. Furthermore, a solid relation with HLA class II alleles, especially HLA-DRB1 alleles, HLA class I alleles also appear to impact on the disposition of this disease. IgAV was intensely related with HLA-DRB1 (Amoli et al., 2001[6]; Lopez-Mejias et al., 2015[150][151], 2018[149]) in the European populations, mostly due to HLA-DR1*0103 (Lopez-Mejias et al., 2015[150][151], 2017[148]).

#### Clinical features

IgAV manifests in 95 % of cases with a skin rash (Nong et al., 2007[179]). Furthermore, the disorder presents with a typical triad of symptoms concerning the musculoskeletal, renal and gastrointestinal systems (Eleftheriou and Brogan, 2009[46]). Less frequently, IgAV can include the respiratory or nervous system. The rash is a symmetrical erythematosus petechial or purpuric rash. The zones of purpura are usually palpable and start on the lower limbs and buttocks (Oni and Sampath, 2019[180]). 

Musculoskeletal involvement is present in 70 %-90 % of patients, presenting as either arthralgia or arthritis. The incidence of arthritis is inferior than arthralgia, and tends to affect 4 or fewer joints, especially those of the lower limb (Trapani et al., 2005[239]). 

Gastrointestinal signs and symptoms may precede the skin manifestations, and typically manifest with colicky abdominal pain and sometimes acute gastrointestinal bleeding (Nong et al., 2007[179]).

Renal involvement, called IgAV nephritis, is usually asymptomatic and microscopic hematuria is the most frequent sign of urinalysis followed by proteinuria without edema (Nong et al., 2007[179]). 

#### Diagnosis

Diagnosis is clinical; rash associated to gastrointestinal, musculoskeletal, or renal symptoms are found in nearly 95 % of patients.

In 2005, a new EULAR/Paediatric Rheumatology European Society (PRES) classification criterion was proposed (Ozen et al., 2006[185]) and certified with the encouragement of the Paediatric Rheumatology International Trials Organization (PRINTO) in 872 cases of IgAV aged ≤ 18 years at diagnosis onset. The EULAR/PRES/PRINTO criteria (Ozen et al., 2010[184]) are based on clinical symptoms and comprise the presence of a vasculitic purpuric rash together with other symptoms and signs with very high sensitivity (100 %) and specificity (87 %) in differentiating IgAV from other vasculitides (Table 4(Tab. 4)).

#### Treatment

The treatment of IgAV is mainly symptom-oriented; actual evidence-based management protocols are still missing for IgAV in childhood. Single-Hub Access for pediatric Rheumatology in Europe (SHARE) initiative (Wulffraat et al., 2013[255]) has been establish to improve protocols for pediatric vasculitis. At present, the use of medications at disease onset in all patients is not supported by clear evidence. Therapy might be needed throughout the acute period for renal involvement and to treat gastrointestinal manifestations. End stage kidney disease is the most important expression and necessitates a period of kidney monitoring of 6-12 months. Guidelines propose using oral steroids for mild forms, steroids plus azathioprine or mycophenolate mofetil or cyclophosphamide for moderate forms and steroids with cyclophosphamide for severe forms (Oni and Sampath, 2019[180]). 

### Hypocomplementemic urticarial vasculitis/Anti-C1q vasculitis

Hypocomplementemic urticarial vasculitis (HUV), also called anti-C1q vasculitis, is an autoimmune condition characterized by long-term urticaria with hypocomplementemia in addition to systemic signs, such as glomerulonephritis, arthritis/arthralgia, uveitis or recurrent abdominal pain (Ozen et al., 2010[184]). Pulmonary manifestations with chronic obstructive pulmonary disease (COPD) are often present and represent an important cause of morbidity and mortality (Schwartz et al., 1982[217]). 

#### Epidemiology

No unique prevalence and incidence data for HUV are available. A Swedish study conducted between 2000 and 2015 in nearly 1.5 million individuals reported only 16 HUV patients, mainly females. In this study, the incidence was 0.7 per million, with no specific age variation. The mean age of onset was 51 years; 5-year survival rate was 92 % and the 10-year survival rate was 83 % (Sjowall et al., 2018[225]). 

#### Etiopathogenesis

The pathophysiology of HUV is not well-defined (Buck et al., 2012[21]). C1q-precipitins (C1q-p) involving IgG autoantibodies bind to the Fc portion of the C1 molecule and form immune complexes thus activating the complement system (Chew and Gatenby, 2007[31]). This cascade upregulates chemokine, cytokine and anaphylatoxin production, which contribute to amplified vascular permeability, chemotaxis of inflammatory cells, and deposition of immune complexes that aggravate tissue damage and edema (Marder et al., 1978[157]). This results in urticaria and/or angioedema and leads to leukocytoclastic vasculitis (Davis and Brewer, 2004[37]; Grotz et al., 2009[79]). 

The exact mechanism of association between HUV and COPD is currently unknown. Anti-C1q autoantibodies have been found in lung disorders and could be accountable for the pulmonary manifestations (Grotz et al., 2009[79]) as C1q precipitins bind to pulmonary alveoli surfactant proteins causing COPD when paired with the vasculitis of pulmonary capillaries and venules (Friskel and Foster, 2000[62]). 

#### Clinical features

The main clinical manifestation of HUV is recurrent urticaria, with skin eruptions that principally affect the trunk, face and upper extremities (Buck et al., 2012[21]). Urticarial rash last 2 to 4 days, then disappears without scarring. Angioedema may also be discovered in patients with involvement of deeper vessels. 

Arthralgia and arthritis are the most common systemic manifestations of HUV, appearing in up to 50 % of patients. The joint pain is usually transient, and joint deformities may occur (Amano et al., 2008[5]). 

Renal presentation is typically minor, but dialysis may be necessary. Proteinuria and hematuria are common following membranous, membranoproliferative, or intra- and extra-capillary glomerulonephritis (Ghamra and Stoller, 2003[67]; Enriquez et al., 2005[47]). 

Lung symptoms includes dyspnea, coughing, hemoptysis, pleural effusion, and COPD; these symptoms occur in about 50 % of HUV patients (Wisnieski and Jones, 1992[254]).

Almost 30 % of HUV cases report gastrointestinal signs, such as pain and nausea, in connection with hepatomegaly, serositis, and splenomegaly (Wisnieski and Jones, 1992[254]). Cases of adenocarcinoma are rare but reported in literature (Buck et al., 2012[21]). 

About 30 % of patients manifest ocular symptoms, typically inflammation of the uveal tract, but even conjunctivitis and episcleritis (Wisnieski et al., 1995[253]). 

Cardiac involvement manifests with valvular abnormalities and congestive heart failure (Wisnieski and Jones, 1992[254]). 

The central nervous system is infrequently affected. Neurological signs may comprise seizure disorder, cranial nerve palsies, axonal neuropathy, mononeuritis, pseudotumor cerebri, aseptic meningitis, and peripheral neuropathy (Davis and Brewer, 2004[37]). 

#### Diagnosis

Skin tissue samples are crucial for the diagnosis of HUV, associated to the presence of anti-C1q antibodies. Other classical laboratory results comprise reduced complement pathway components (C1q, C3 and C4) and/or low levels of complement function in serum. 

Pathology reveals a vasculitis of small blood vessels, often associated to leukocytoclasia and perivascular infiltrates made of neutrophils. The discovery of standard complement components with immunofluorescence microscopy in biopsies further strengthens the diagnosis of HUV (Buck et al., 2012[21]). 

In 1982, Schwartz et al. recognized the diagnostic criteria for HUV (Schwartz et al., 1982[217]). Two major criteria (hypocomplementemia and recurrent urticaria for more than 6 months) and at least two minor criteria (skin biopsy, ocular inflammation, arthralgias or arthritis, glomerulonephritis, abdominal pain, and positive C1q-p test by immunodiffusion with decreased C1q level) are necessary for the diagnosis (Filosto et al., 2009[56]; Jara et al., 2009[105]). 

Criteria for exclusion are cryoglobulinemia (cryocrit > 1 %), hepatitis B virus antigenemia, elevated titer of Sm antibodies or anti-double-stranded DNA antibody (dsDNA), deficiency of C1 esterase levels, and a high titer of ANA (Siegert et al., 1991[222]; Wisnieski et al., 1995[253]; Aydogan et al., 2006[12]). 

#### Treatment

Patients whose serum complement levels remain normal usually present with a self-limited disorder and necessitate little or no treatment. Antihistamine is the preferred drug to treat patients with only cutaneous manifestations (Wisnieski et al., 1995[253]). 

Nonsteroidal anti-inflammatory drugs may be used for symptomatic relief of joint pain (Aydogan et al., 2006[12]). 

Life-threatening involvement of the pulmonary or other tissues require specific treatments and intense immunosuppression. Consequently, treatment management in HUV must be individualized based on patient's clinical manifestations. 

Cytotoxic agents used in HUV include cyclophosphamide, mycophenolate mofetil, azathioprine, cyclosporine A, and methotrexate, alone or in combination with prednisolone. Rituximab should be contemplated if symptoms are refractory (Schwartz et al., 1982[217]; Fortson et al., 1986[59]; Mehregan et al., 1992[163]; Wisnieski et al., 1995[253]; Saigal et al., 2003[206]). Plasmapheresis and intravenous immunoglobulin have been advised as to be pondered in those patients with rapid decline of kidney function (Balsam et al., 2008[15]). 

## Variable Vessel Vasculitis (VVV)

### Behçet's disease 

Behçet's disease (BD) is a systemic vasculitis with phases of exacerbation and remission of unknown etiology. 

The disease was first described by the Turkish dermatologist Hulusi Behçet in 1937 (Sakane et al., 1999[207]; Alpsoy, 2016[4]), andis characterized by recurrent occurrence of oral aphthous ulcers, ocular lesions and genital ulcers; additional characteristics comprise cardiovascular, neurological, dermatological, and gastrointestinal manifestations (Greco et al., 2018[74]). 

#### Epidemiology

BD has been studied in many populations with large differences; the highest rates are found in Eastern Mediterranean countries and China suggesting a role for environmental or genetic factors (Verity et al., 1999[246]).

Maldini et al. in their meta-analysis found the geographical discrepancy in the prevalence of BD. The worldwide prevalence was 10.3/100,000 people. Pooled prevalence are 119.8/100,000 inhabitants in Turkey, 31.8/100,000 inhabitants in the Middle East, 4.5/100,000 in Asia and 3.3/100,000 inhabitants in Europe (Maldini et al., 2018[156]). In European countries, higher prevalence is found in southern Europe, thus advocating that BD is sporadic amongst northern European ancestry populations.

The genetic predisposition of BD has been examined, and an association with HLA-B*51 has been reported (Wallace, 2014[248]). 

#### Etiopathogenesis

Higher prevalence in precise familial and geographic areas suggest a central etiopathogenetic role for genetic factors. Familial aggregation has been seen in families of Turkish (18 %-20 %), Korean (15 %-17 %), and Israeli origin (10 %-13 %) (Zouboulis, 1999[263]; Fietta, 2005[54]).

De Menthon et al. in their meta-analysis studied the association between BD and HLA-B51/B5, and sustained that this allele is a primary and causal risk determinant for BD (de Menthon et al., 2009[38]). 

A recent study recognized that common variants of the IL-10 and encoding IL-23 receptor (IL23R) and encoding IL-12 receptor beta (IL12B2) genes were powerfully linked with BD (Morton et al., 2016[170]). IL-23, proinflammatory cytokine, promotes Th17 production, rises generation of inflammatory cytokines, and improves the proliferation of IL-23 p19 mRNA in erythema nodosum-like skin lesions in patients with acute BD (Lew et al., 2008[141]). IL-10 prevents the release of proinflammatory cytokines (Shim et al., 2011[220]). 

Bacteria and viruses have long been hypothesized as probable causes of BD, particularly *Streptococcus sanguinis* and *Herpes simplex* virus (HSV) type 1. However, so far, no exact microorganism has been demonstrated as causal trigger for BD. Modest oral health and tonsillitis are more frequent in patients with BD; for this reason, the responsibility of *Streptococcus sanguinis* has been widely examined in the etiopathogenesis of the BD (Kaneko et al., 2008[115]). A different microbiome pattern was described in the gut of Italian patients (Consolandi et al., 2015[33]) and in another study the saliva of Turkish patients (Coit et al., 2016[32]).

HSV type 1 can be found in intestinal ulcers, saliva, and genital ulcers by polymerase chain reaction (PCR) in BD cases compared with healthy controls. Lee et al. found HSV DNA in 36.4 % of BD patients with oral ulceration and in 42.4 % of patients without oral ulceration at the time of testing (Lee et al., 1996[138]). Nevertheless, there is no strong evidence supporting the responsibility of single microorganisms as specific etiologic agents of BD.

#### Clinical features

Oral aphthae appear in 98 % of patients and are the hallmark of the disease following the International Study Group for Behçet's Disease (1990[101]) criteria.

The characteristic aphthae are rounded with an erythematous, sharp and elevated margin. The lesions are painful and are usually of 1 to 3 cm in diameter (Hatemi et al., 2018[85][86]).

Genital aphthae are less frequent than oral lesions (60 % to 65 % of patients) but still are very advocating of the diagnosis of this vasculitis. Genital lesions are present on the vulva and vagina and on the scrotum. They appear similar to the oral ulcers but frequently deeper and larger. Other skin lesions, such as erythema nodosum, acneiform nodules, pseudofolliculitis, and papulopustular lesions, are less commonly reported.

Eye mentation happens in 30 %-70 % of patients with BD and almost 25 % of patients will become blind despite cure. Ocular involvement is characterized by a relapsing bilateral non-granulomatous uveitis (Mendes et al., 2009[165]). 

Vascular lesions are described both in arteries and veins, although the latter are more frequently involved. Venous thrombosis happens in about 30 % of patients, while arterial involvement is appreciated in just 3 %-5 % of patients (Saadoun et al., 2012[204]). 

Arthralgia occurs in nearly 45 % of patients and it can be the first clinical manifestation; the knees and ankles are the most convoluted joint.

Neurological signs are seen in 20 %-40 % of patients (Akman-Demir et al., 1999[3]). The involvement of the central nervous system comprises non-parenchymal and parenchymal lesions (neuro-Behçet's disease). The spectrum of symptoms ranges from headache, hemiplegia, cranial nerve palsies or meningitis. Rarely, psychiatric manifestations may occur, and patients can development personality changes.

Gastrointestinal involvement in BD cases manifests with abdominal pain, nausea and diarrhea. The clinical manifestations are very similar to that of inflammatory bowel disease, and differential diagnosis is often challenging (Hatemi et al., 2018[85][86]). 

The common audio-vestibular alterations in BD are disequilibrium and hearing loss. Sensorineural hearing loss is the more frequent audiological manifestation and is described as mainly bilateral sensorineural hearing loss that predominantly affects high frequencies. Vertigo is the other common audiological symptom (Ralli et al., 2018[191][192]). 

#### Diagnosis

Currently there is not a gold standard for the diagnosis of BD, therefore anamnesis and clinical examination play a central role.

For the diagnosis, a minimum of two major manifestations are needed. The most common manifestations are aphthous-like ulcerations of the genital and oral mucosa and uveitis (Greco et al., 2018[74]). Additional clinical symptoms that can be associated with the disease are pulmonary, cardiovascular, audio-vestibular, dermatological, musculoskeletal, gastrointestinal, and nervous disorders. 

Similar to other vasculitides, there are no specific clinical or laboratorial findings of BD. An International Study Group (ISG) for BD was reunited in 1990 to produce a protocol of criteria for the diagnosis of BD that included recurrent oral ulcers plus 2 other signs (1990[101]) (Table 5(Tab. 5)). 

The pathergy test is a sensitive tool for BD diagnosis and consists in a non-specific skin hyperreactivity following intradermal puncture (Ozdemir et al., 2007[183]); however, its positivity varies with geographical area, ranging from 60 % positive cases in Middle Eastern patients to 5 % in Caucasian patients, which significantly decreases its diagnostic values in populations with low positivity (Androudi, 2006[7]). 

Diagnosis of patients with BD is just sustained by clinical criteria; for this reason, differential diagnosis is very important. Oral ulcerations might appear in 30 %-40 % of the general population and are not exclusive of BD, while bipolar ulcerations are typical of BD. Oral ulcerations can similarly be linked to hemopathy, Crohn's disease, HIV, Crohn's disease, Lupus Erythematous, bullous dermatosis and vitamin deficit. Sarcoidosis, Vogt-Koyanagi Harada (Evereklioglu, 2005[50]), Cogan syndrome (D'Aguanno et al., 2018[36]) and Crohn's disease must be excluded when ocular manifestations occur. Arterial alterations may mimic TA. Neuro-Behçet's disease should be differentiated from Susac syndrome or multiple sclerosis (Greco et al., 2014[73]). Differential diagnosis with sudden sensorineural hearing loss and Meniere's disease should be considered for audio-vestibular symptoms (Fusconi et al., 2012[63]).

#### Treatment

In 2018, a task force has updated the 2008 EULAR recommendations for the treatment of BD (Hatemi et al., 2018[85]). Colchicine is the first-line therapy for mucocutaneous manifestations; nevertheless, there is no indication of its efficiency in oral ulcers (Yurdakul et al., 2001[260]). Interferon (IFN)-α-2a, azathioprine and TNF-α inhibitors are advised for intractable mucocutaneous lesions (Hatemi et al., 2018[85][86]).

Grayson et al. recently described that 200mg/day of anakinra had an acceptable safety profile and was effective in the therapy of resistant oral and genital ulcers (Grayson et al., 2017[72]). 

In case of ocular involvement, first-line therapy consists in systemic corticosteroids. Cyclosporine-A, TNF-α inhibitors, or IFN-α-2a can also be used in refractory patients (Yurdakul et al., 2001[260]).

There are no randomized controlled trials for the treatment of main vascular involvement in BD and, consistently with EULAR recommendations, only immunosuppressive drugs are recommended. 

No clear evidence is available for the treatment of neurologic involvement in Neuro-Behçet's disease. High-dose intravenous methylprednisolone has been recommended for the acute phases, followed by azathioprine and oral corticosteroids (Akman-Demir et al., 2011[2]).

5-Aminosalicylic acid (ASA) derivatives with or without corticosteroids have been suggested for minor gastrointestinal involvement in BD patients (Jung et al., 2012[111][112]). Hatemi et al. found that Thalidomide and TNF-α inhibitors might similarly be utilized patients with gastrointestinal involvement that were refractory (Hatemi et al., 2015[87]). 

### Cogan's syndrome

Cogan's syndrome (CS) is an uncommon systemic vasculitis of unknown origin first described in 1934. Typical signs are ocular and audio-vestibular symptoms. The spectrum of ocular manifestations include non-syphilitic interstitial keratitis (IK), uveitis, retinal vasculitis, conjunctivitis, retinal vasculitis, scleritis, tinnitus hearing loss and vertigo (D'Aguanno et al., 2018[36]). An infectious factor appears to be the possible trigger (Kessel et al., 2014[121]), although in the current years the function of autoimmunity has increased interest in the pathogenesis of CS (Jung et al., 2016[109]). Currently, the etiology of CS is still unclear and further research is needed.

#### Epidemiology

CS is a uncommon disease which mainly affects young adults (Iliescu et al., 2015[100]). The beginning of the disorder is usually in individuals younger than 30 years with no gender preference. In the literature less than 250 cases have been reported and almost all of them are Caucasian patients (Iliescu et al., 2015[100]), while CS is particularly uncommon in Arabic and Middle Eastern areas (Gaubitz et al., 2001[66]; Cundiff et al., 2006[35]).

#### Etiopathogenesis

The pathogenetic basis of CS still remains unknown; a role for upper respiratory tract infections has been hypothesized as they precede the onset of CS in 50 % of patients (Vollertsen et al., 1986[247]). 

Chlamydial infection is the most studied bacterial infection because it is able of eluding host defence and can provoke chronic infection. Nevertheless, a direct association among altered Chlamydia species and CS has not been established yet. 

Furthermore, a viral involvement has been suggested, because a cross-reaction has been shown between the antibodies against the Cogan peptide and proteins of the Reovirus *type* III (Lunardi et al., 2002[152]).

The role of the immune system was proposed by the discovery of varied autoantibodies, clinical elements suggestive of collagen disease, and manifestation of vasculitis and polyarteritis nodosa. Cogan peptides' sequence has homology with CD148 and Connexin 26, which are shown in the inner ear and on endothelial cells (Lunardi et al., 2002[152]). 

Connexin 26 appears similar to Connexin 50 and Connexin 43, gap junction proteins described in epithelium and corneal fibroblasts. Since non-syphilitic IK is an additional typical clinical sign of CS, this could clarify the eye manifestations. The antigen is moreover present on glial and nerve cells and could elucidate the different symptoms of CS, comprising the neurological alterations (Autschbach et al., 1999[11]). 

#### Clinical features

The infection of the upper respiratory tract is often the first symptom of CS. Moreover, the two main manifestation are ear and eye clinical features.

The spectrum of symptoms related to ocular involvement in CS is variable; IK is the most frequent, followed by retinal vascular disease, scleritis, episcleritis, uveitis, conjunctivitis, papilledema and exophthalmos (Garcia Berrocal et al., 1999[64]).

The audio-vestibular involvement is very similar to Meniere's disease, with an acute onset characterized by vertigo, instability, nausea, vomiting, tinnitus, and - most important - rapidly progressive hearing loss that leads to deafness in a period of 1-3-months (Ralli et al., 2018[191][192]). Audiometry usually shows sensorineural hearing loss for all frequencies; however, the hearing loss is more evident for high frequencies (Ralli et al., 2018[191][192]).

Associated cardiovascular, neurological and gastrointestinal symptoms are often present. Cardiac involvement is principally represented by aortic insufficiency, showed in almost 15 % of patients; nearly half of them necessitate valve substitution. Gastrointestinal symptoms include diarrhea, rectal bleeding, abdominal pain, hepatomegaly and splenomegaly (Grasland et al., 2004[71]). Neurologic signs are not specific; the spectrum of manifestations range from headache to coma (Grasland et al., 2004[71]).

The risk of systemic vasculitis has to be carefully studied at any stage of CS. Histologic alteration are similar to polyarteritis nodosa, including conspicuous penetration of large veins and muscular artery walls with neutrophils and lymphocytes (Espinoza and Prost, 2015[48]).

#### Diagnosis

Currently there is not a hallmark for the diagnosis of CS. The diagnosis is primarily clinical and is based on audio-vestibular symptoms, eye involvement and good response to corticosteroid. Prevalent additional criteria include fever, weight loss, fatigue, headache, lymphadenopathy, and increased blood concentration of systemic inflammatory markers (D'Aguanno et al., 2018[36]). 

The diagnosis is mainly focused on the audio-vestibular signs and symptoms, the ocular manifestations and nonreactive serological tests for syphilis; clinical diagnostic tests include pure tone audiometry, echocardiography, doppler test, and angiography, with a multidisciplinary approach that includes ophthalmologists, otolaryngologists and internists (Iliescu et al., 2015[100]).

There are numerous disorders that can be suggested in the differential diagnosis of CS: congenital syphilis, Vogt-Koyanagi-Harada syndrome (Greco et al., 2013[76]), Susac syndrome, and other systemic diseases such as polyarteritis nodosa, TA and Wegener's granulomatosis (Gaubitz et al., 2001[66]).

#### Treatment

Management of CS is tailored on the status of the disease and on patient's symptoms. IK usually responds well to steroid eye drops or local atropine, while in case of audio-vestibular symptoms prompt therapy with systemic steroids (1-2 mg/kg/day prednisolone) is needed (Gaubitz et al., 2001[66]). The corticosteroid protocol should be rapidly discontinued if there is no improvement within 2 weeks; contrarily, if improvements are found, steroids must be slowly tapered over 2-6 months (Grasland et al., 2004[71]).

No therapy has been demonstrated effective to improve audio-vestibular alterations when they do not respond to steroid treatment. In these cases, immunosuppressive drugs are added. The most used drugs are cyclosporine A, azathioprine, and methotrexate. Cochlear implant should be considered in patients that develop profound sensorineural hearing loss (Gaubitz et al., 2001[66]).

The treatment has to be more aggressive when the vasculitis is extensive; in these cases cytotoxic agents are recommended (Gaubitz et al., 2001[66]).

A new therapeutic option for CS are the TNF-α blockers (Fricker et al., 2007[61]). Infliximab appears to be efficient in provoking remission in patients with therapy resistant CS (Fricker et al., 2007[61]). Rituximab might support in avoiding deafness or cochlear implantation in complicated patients; however, it is not suggested as a first line therapy (Orsoni et al., 2010[182]).

The progress of CS is irregular. Patients with eye and ear manifestations have a good prognosis and quality of life, while cases with extensive vasculitis have more complications and a worse prognosis.

## Conclusions

Vasculitides are uncommon conditions that can lead to different complex disorders potentially involving any organ and system. Vasculitides can be classified based on primarily affected vessel sizes (large, medium, small), clinical phenotypes, underlying causes, or histological patterns. The pathogenetic mechanisms of systemic vasculitides are still partly unknown. To date, there are no single pathognomonic tests and no specific diagnostic criteria for most systemic vasculitides; therefore, often their diagnosis is made after having ruled out other mimicking conditions. However, a precise and timely diagnosis is of utmost importance for these conditions, as it allows to initiate proper treatment and avoid progression to multiorgan failure with irreversible damage. 

## Declarations

The authors declare that they have no conflict of interest.

## Funding

None.

## Figures and Tables

**Table 1:**
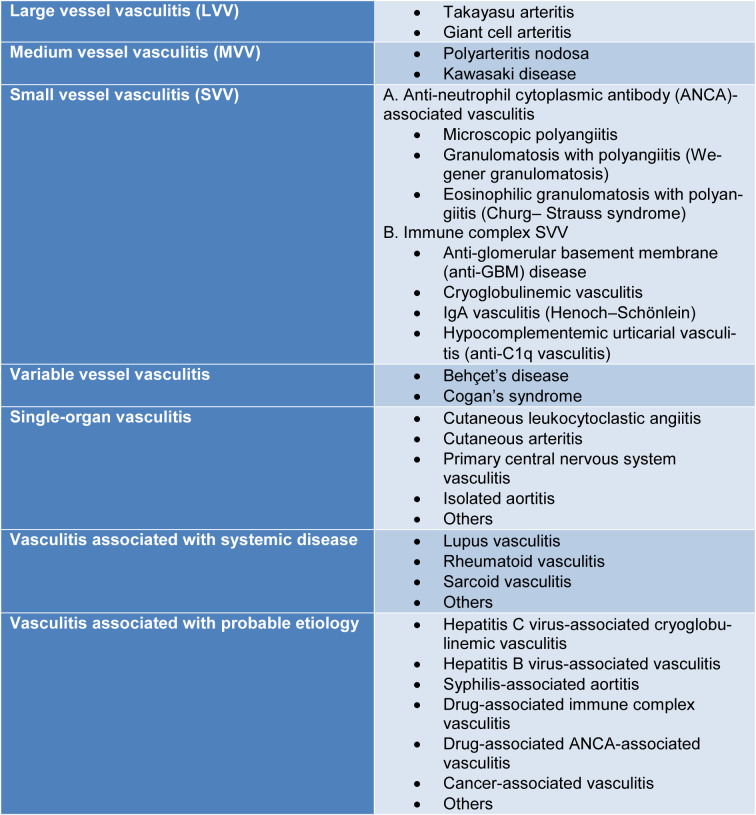
Chapel Hill Consensus Conference 2012 Classification criteria

**Table 2:**
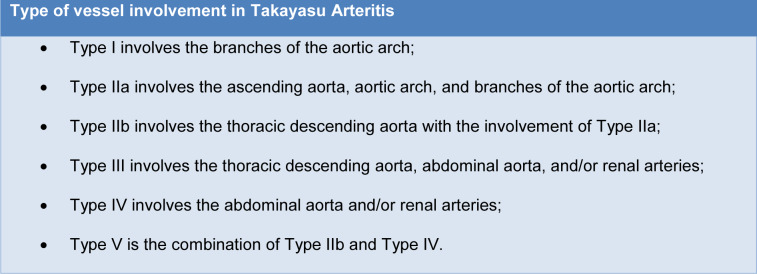
Different types of vessel involvement based on conventional angiographic findings published by the International Conference on Takayasu Arteritis in 1994

**Table 3:**
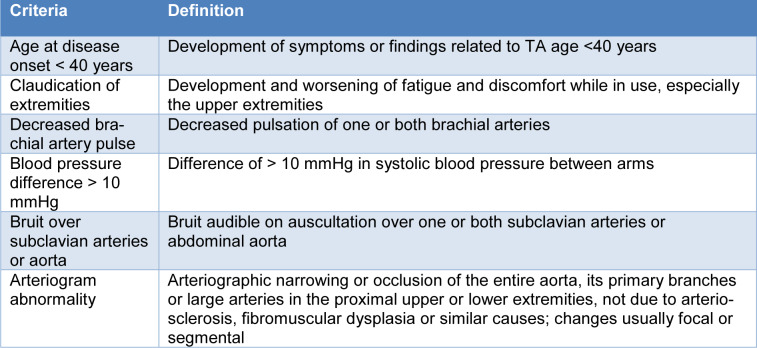
The 1990 American College of Rheumatology Classification criteria for Takayasu arteritis. For classification purposes, a patient is said to have Takayasu arteritis if at least three of the six criteria are present (from Arend et al., 1990).

**Table 4:**
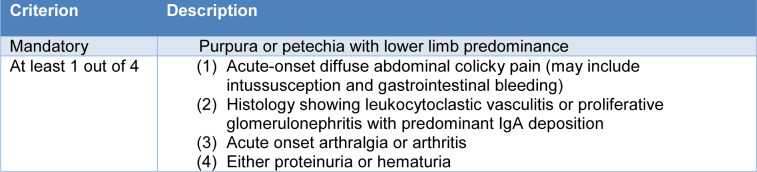
European League Against Rheumatism (EULAR) / Paediatric Rheumatology European Society (PRES) / Paediatric Rheumatology International Trials Organization (PRINTO) classification criteria for childhood IgA vasculitis

**Table 5:**
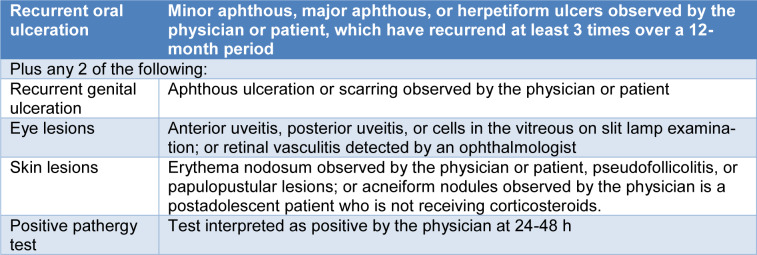
International Study Group (ISG) criteria for the diagnosis of Behcet's disease

## References

[1] Agard C, Barrier JH, Dupas B, Ponge T, Mahr A, Fradet G (2008). Aortic involvement in recent-onset giant cell (temporal) arteritis: a case-control prospective study using helical aortic computed tomodensitometric scan. Arthritis Rheum.

[2] Akman-Demir G, Saip S, Siva A (2011). Behcet's disease. Curr Treat Options Neurol.

[3] Akman-Demir G, Serdaroglu P, Tasci B (1999). Clinical patterns of neurological involvement in Behcet's disease: evaluation of 200 patients. The Neuro-Behcet Study Group. Brain.

[4] Alpsoy E (2016). Behcet's disease: A comprehensive review with a focus on epidemiology, etiology and clinical features, and management of mucocutaneous lesions. J Dermatol.

[5] Amano H, Furuhata N, Tamura N, Tokano Y, Takasaki Y (2008). Hypocomplementemic urticarial vasculitis with Jaccoud's arthropathy and valvular heart disease: case report and review of the literature. Lupus.

[6] Amoli MM, Thomson W, Hajeer AH, Calvino MC, Garcia-Porrua C, Ollier WE (2001). HLA-DRB1*01 association with Henoch-Schonlein purpura in patients from northwest Spain. J Rheumatol.

[7] Androudi S (2006). Current concepts in the etiology and treatment of Behcet disease. Surv Ophthalmol.

[8] Arend WP, Michel BA, Bloch DA, Hunder GG, Calabrese LH, Edworthy SM (1990). The American College of Rheumatology 1990 criteria for the classification of Takayasu arteritis. Arthritis Rheum.

[9] Arnaud L, Haroche J, Mathian A, Gorochov G, Amoura Z (2011). Pathogenesis of Takayasu's arteritis: a 2011 update. Autoimmun Rev.

[10] Audemard-Verger A, Terrier B, Dechartres A, Chanal J, Amoura Z, Le Gouellec N (2017). Characteristics and management of IgA vasculitis (Henoch-Schonlein) in adults: Data from 260 patients included in a French multicenter retrospective survey. Arthritis Rheumatol.

[11] Autschbach F, Palou E, Mechtersheimer G, Rohr C, Pirotto F, Gassler N (1999). Expression of the membrane protein tyrosine phosphatase CD148 in human tissues. Tissue Antigens.

[12] Aydogan K, Karadogan SK, Adim SB, Tunali S (2006). Hypocomplementemic urticarial vasculitis: a rare presentation of systemic lupus erythematosus. Int J Dermatol.

[13] Baker AL, Gauvreau K, Newburger JW, Sundel RP, Fulton DR, Jenkins KJ (2003). Physical and psychosocial health in children who have had Kawasaki disease. Pediatrics.

[14] Balow JE (1985). Renal vasculitis. Kidney Int.

[15] Balsam L, Karim M, Miller F, Rubinstein S (2008). Crescentic glomerulonephritis associated with hypocomplementemic urticarial vasculitis syndrome. Am J Kidney Dis.

[16] Biesenbach P, Kain R, Derfler K, Perkmann T, Soleiman A, Benharkou A (2014). Long-term outcome of anti-glomerular basement membrane antibody disease treated with immunoadsorption. PLoS One.

[17] Bolton WK, Innes DJ, Sturgill BC, Kaiser DL (1987). T-cells and macrophages in rapidly progressive glomerulonephritis: clinicopathologic correlations. Kidney Int.

[18] Bourgarit A, Le Toumelin P, Pagnoux C, Cohen P, Mahr A, Le Guern V (2005). Deaths occurring during the first year after treatment onset for polyarteritis nodosa, microscopic polyangiitis, and Churg-Strauss syndrome: a retrospective analysis of causes and factors predictive of mortality based on 595 patients. Medicine (Baltimore).

[19] Brekke LK, Diamantopoulos AP, Fevang BT, Abetamus J, Espero E, Gjesdal CG (2017). Incidence of giant cell arteritis in Western Norway 1972-2012: a retrospective cohort study. Arthritis Res Ther.

[20] Brouet JC, Clauvel JP, Danon F, Klein M, Seligmann M (1974). Biologic and clinical significance of cryoglobulins. A report of 86 cases. Am J Med.

[21] Buck A, Christensen J, McCarty M (2012). Hypocomplementemic urticarial vasculitis syndrome: a case report and literature review. J Clin Aesthet Dermatol.

[22] Burns JC, Franco A (2015). The immunomodulatory effects of intravenous immunoglobulin therapy in Kawasaki disease. Expert Rev Clin Immunol.

[23] Burns JC, Herzog L, Fabri O, Tremoulet AH, Rodo X, Uehara R (2013). Seasonality of Kawasaki disease: a global perspective. PLoS One.

[24] Cacoub P, Comarmond C, Domont F, Savey L, Saadoun D (2015). Cryoglobulinemia vasculitis. Am J Med.

[25] Calvino MC, Llorca J, Garcia-Porrua C, Fernandez-Iglesias JL, Rodriguez-Ledo P, Gonzalez-Gay MA (2001). Henoch-Schonlein purpura in children from northwestern Spain: a 20-year epidemiologic and clinical study. Medicine (Baltimore).

[26] Carmona FD, Mackie SL, Martin JE, Taylor JC, Vaglio A, Eyre S (2015). A large-scale genetic analysis reveals a strong contribution of the HLA class II region to giant cell arteritis susceptibility. Am J Hum Genet.

[27] Carmona FD, Vaglio A, Mackie SL, Hernandez-Rodriguez J, Monach PA, Castaneda S (2017). A genome-wide association study identifies risk alleles in plasminogen and P4HA2 associated with giant cell arteritis. Am J Hum Genet.

[28] Cavalli G, Tomelleri A, Di Napoli D, Baldissera E, Dagna L (2018). Prevalence of Takayasu arteritis in young women with acute ischemic heart disease. Int J Cardiol.

[29] Chacko JG, Chacko JA, Salter MW (2015). Review of giant cell arteritis. Saudi J Ophthalmol.

[30] Chen SF, Wang FM, Li ZY, Yu F, Chen M, Zhao MH (2018). Myeloperoxidase influences the complement regulatory activity of complement factor H. Rheumatology (Oxford).

[31] Chew GY, Gatenby PA (2007). Inflammatory myositis complicating hypocomplementemic urticarial vasculitis despite on-going immunosuppression. Clin Rheumatol.

[32] Coit P, Mumcu G, Ture-Ozdemir F, Unal AU, Alpar U, Bostanci N (2016). Sequencing of 16S rRNA reveals a distinct salivary microbiome signature in Behcet's disease. Clin Immunol.

[33] Consolandi C, Turroni S, Emmi G, Severgnini M, Fiori J, Peano C (2015). Behcet's syndrome patients exhibit specific microbiome signature. Autoimmun Rev.

[34] Csernok E, Lamprecht P, Gross WL (2006). Diagnostic significance of ANCA in vasculitis. Nat Clin Pract Rheumatol.

[35] Cundiff J, Kansal S, Kumar A, Goldstein DA, Tessler HH (2006). Cogan's syndrome: a cause of progressive hearing deafness. Am J Otolaryngol.

[36] D'Aguanno V, Ralli M, de Vincentiis M, Greco A (2018). Optimal management of Cogan's syndrome: a multidisciplinary approach. J Multidiscip Healthc.

[37] Davis MD, Brewer JD (2004). Urticarial vasculitis and hypocomplementemic urticarial vasculitis syndrome. Immunol Allergy Clin North Am.

[38] de Menthon M, Lavalley MP, Maldini C, Guillevin L, Mahr A (2009). HLA-B51/B5 and the risk of Behcet's disease: a systematic review and meta-analysis of case-control genetic association studies. Arthritis Rheum.

[39] De Virgilio A, Greco A, Magliulo G, Gallo A, Ruoppolo G, Conte M (2016). Polyarteritis nodosa: A contemporary overview. Autoimmun Rev.

[40] Demir S, Sonmez HE, Ozen S (2019). Vasculitis: Decade in Review. Curr Rheumatol Rev.

[41] Desbois AC, Cacoub P, Saadoun D (2019). Cryoglobulinemia: An update in 2019. Joint Bone Spine.

[42] Di Santo M, Stelmaszewski EV, Villa A (2018). Takayasu arteritis in paediatrics. Cardiol Young.

[43] Dick J, Gan PY, Ford SL, Odobasic D, Alikhan MA, Loosen SH (2018). C5a receptor 1 promotes autoimmunity, neutrophil dysfunction and injury in experimental anti-myeloperoxidase glomerulonephritis. Kidney Int.

[44] Dietz SM, van Stijn D, Burgner D, Levin M, Kuipers IM, Hutten BA (2017). Dissecting Kawasaki disease: a state-of-the-art review. Eur J Pediatr.

[45] Elakabawi K, Lin J, Jiao F, Guo N, Yuan Z (2020). Kawasaki disease: global burden and genetic background. Cardiol Res.

[46] Eleftheriou D, Brogan PA (2009). Vasculitis in children. Best Pract Res Clin Rheumatol.

[47] Enriquez R, Sirvent AE, Amoros F, Perez M, Matarredona J, Reyes A (2005). Crescentic membranoproliferative glomerulonephritis and hypocomplementemic urticarial vasculitis. J Nephrol.

[48] Espinoza GM, Prost A (2015). Cogan's syndrome and other ocular vasculitides. Curr Rheumatol Rep.

[49] Evans JM, Hunder GG (2000). Polymyalgia rheumatica and giant cell arteritis. Rheum Dis Clin North Am.

[50] Evereklioglu C (2005). Current concepts in the etiology and treatment of Behcet disease. Surv Ophthalmol.

[51] Farh KK, Marson A, Zhu J, Kleinewietfeld M, Housley WJ, Beik S (2015). Genetic and epigenetic fine mapping of causal autoimmune disease variants. Nature.

[52] Faverio P, Bonaiti G, Bini F, Vaghi A, Pesci A (2018). Mepolizumab as the first targeted treatment for eosinophilic granulomatosis with polyangiitis: a review of current evidence and potential place in therapy. Ther Clin Risk Manag.

[53] Field J, Shahijanian F, Schibeci S, Australia and New Zealand MS Genetics Consortium (ANZgene), Johnson L, Gresle M, (2015). The MS risk allele of CD40 is associated with reduced cell-membrane bound expression in antigen presenting cells: implications for gene function. PLoS One.

[54] Fietta P (2005). Behcet's disease: familial clustering and immunogenetics. Clin Exp Rheumatol.

[55] Filer AD, Gardner-Medwin JM, Thambyrajah J, Raza K, Carruthers DM, Stevens RJ (2003). Diffuse endothelial dysfunction is common to ANCA associated systemic vasculitis and polyarteritis nodosa. Ann Rheum Dis.

[56] Filosto M, Cavallaro T, Pasolini G, Broglio L, Tentorio M, Cotelli M (2009). Idiopathic hypocomplementemic urticarial vasculitis-linked neuropathy. J Neurol Sci.

[57] Fitzcharles MA, Esdaile JM (1990). Atypical presentations of polymyalgia rheumatica. Arthritis Rheum.

[58] Foroozan R, Deramo VA, Buono LM, Jayamanne DG, Sergott RC, Danesh-Meyer H (2003). Recovery of visual function in patients with biopsy-proven giant cell arteritis. Ophthalmology.

[59] Fortson JS, Zone JJ, Hammond ME, Groggel GC (1986). Hypocomplementemic urticarial vasculitis syndrome responsive to dapsone. J Am Acad Dermatol.

[60] Freire Ade L, Bertolo MB, de Pinho AJ, Samara AM, Fernandes SR (2004). Increased serum levels of interleukin-8 in polyarteritis nodosa and Behcet's disease. Clin Rheumatol.

[61] Fricker M, Baumann A, Wermelinger F, Villiger PM, Helbling A (2007). A novel therapeutic option in Cogan diseases? TNF-alpha blockers. Rheumatol Int.

[62] Friskel E, Foster R (2000). A 37-year-old man with severe COPD, rash, and conjunctivitis. Chest.

[63] Fusconi M, Chistolini A, de Virgilio A, Greco A, Massaro F, Turchetta R (2012). Sudden sensorineural hearing loss: a vascular cause? Analysis of prothrombotic risk factors in head and neck. Int J Audiol.

[64] Garcia Berrocal JR, Vargas JA, Vaquero M, Ramon y Cajal S, Ramirez-Camacho RA (1999). Cogan's syndrome: an oculo-audiovestibular disease. Postgrad Med J.

[65] Gardner-Medwin JM, Dolezalova P, Cummins C, Southwood TR (2002). Incidence of Henoch-Schonlein purpura, Kawasaki disease, and rare vasculitides in children of different ethnic origins. Lancet.

[66] Gaubitz M, Lubben B, Seidel M, Schotte H, Gramley F, Domschke W (2001). Cogan's syndrome: organ-specific autoimmune disease or systemic vasculitis? A report of two cases and review of the literature. Clin Exp Rheumatol.

[67] Ghamra Z, Stoller JK (2003). Basilar hyperlucency in a patient with emphysema due to hypocomplementemic urticarial vasculitis syndrome. Respir Care.

[68] Goel R, Kabeerdoss J, Ram B, Prakash JA, Babji S, Nair A (2017). Serum cytokine profile in Asian Indian patients with Takayasu arteritis and its association with disease activity. Open Rheumatol J.

[69] Gonzalez-Gay MA, Garcia-Porrua C (2001). Epidemiology of the vasculitides. Rheum Dis Clin North Am.

[70] Gonzalez-Gay MA, Lopez-Mejias R, Pina T, Blanco R, Castaneda S (2018). IgA Vasculitis: genetics and clinical and therapeutic management. Curr Rheumatol Rep.

[71] Grasland A, Pouchot J, Hachulla E, Bletry O, Papo T, Vinceneux P (2004). Typical and atypical Cogan's syndrome: 32 cases and review of the literature. Rheumatology (Oxford).

[72] Grayson PC, Yazici Y, Merideth M, Sen HN, Davis M, Novakovich E (2017). Treatment of mucocutaneous manifestations in Behcet's disease with anakinra: a pilot open-label study. Arthritis Res Ther.

[73] Greco A, De Virgilio A, Gallo A, Fusconi M, Turchetta R, Tombolini M (2014). Susac's syndrome - pathogenesis, clinical variants and treatment approaches. Autoimmun Rev.

[74] Greco A, De Virgilio A, Ralli M, Ciofalo A, Mancini P, Attanasio G (2018). Behcet's disease: New insights into pathophysiology, clinical features and treatment options. Autoimmun Rev.

[75] Greco A, De Virgilio A, Rizzo MI, Tombolini M, Gallo A, Fusconi M (2015). Kawasaki disease: an evolving paradigm. Autoimmun Rev.

[76] Greco A, Fusconi M, Gallo A, Turchetta R, Marinelli C, Macri GF (2013). Vogt-Koyanagi-Harada syndrome. Autoimmun Rev.

[77] Greco A, Marinelli C, Fusconi M, Macri GF, Gallo A, De Virgilio A (2016). Clinic manifestations in granulomatosis with polyangiitis. Int J Immunopathol Pharmacol.

[78] Greco A, Rizzo MI, De Virgilio A, Gallo A, Fusconi M, Ruoppolo G (2015). Churg-Strauss syndrome. Autoimmun Rev.

[79] Grotz W, Baba HA, Becker JU, Baumgartel MW (2009). Hypocomplementemic urticarial vasculitis syndrome: an interdisciplinary challenge. Dtsch Arztebl Int.

[80] Gu B, Magil AB, Barbour SJ (2016). Frequently relapsing anti-glomerular basement membrane antibody disease with changing clinical phenotype and antibody characteristics over time. Clin Kidney J.

[81] Gudbrandsson B, Molberg O, Garen T, Palm O (2017). Prevalence, incidence, and disease characteristics of Takayasu arteritis by ethnic background: data from a large, population-based cohort resident in southern Norway. Arthritis Care Res (Hoboken).

[82] Guillevin L, Pagnoux C, Seror R, Mahr A, Mouthon L, Le Toumelin P (2011). The Five-Factor Score revisited: assessment of prognoses of systemic necrotizing vasculitides based on the French Vasculitis Study Group (FVSG) cohort. Medicine (Baltimore).

[83] Gulati K, McAdoo SP (2018). Anti-glomerular basement membrane disease. Rheum Dis Clin North Am.

[84] Hata A, Noda M, Moriwaki R, Numano F (1996). Angiographic findings of Takayasu arteritis: new classification. Int J Cardiol.

[85] Hatemi G, Christensen R, Bang D, Bodaghi B, Celik AF, Fortune F (2018). 2018 update of the EULAR recommendations for the management of Behcet's syndrome. Ann Rheum Dis.

[86] Hatemi G, Seyahi E, Fresko I, Talarico R, Hamuryudan V (2018). One year in review 2018: Behcet's syndrome. Clin Exp Rheumatol.

[87] Hatemi I, Hatemi G, Pamuk ON, Erzin Y, Celik AF (2015). TNF-alpha antagonists and thalidomide for the management of gastrointestinal Behcet's syndrome refractory to the conventional treatment modalities: a case series and review of the literature. Clin Exp Rheumatol.

[88] Hayreh SS, Podhajsky PA, Zimmerman B (1998). Ocular manifestations of giant cell arteritis. Am J Ophthalmol.

[89] Hayreh SS, Zimmerman B (2003). Visual deterioration in giant cell arteritis patients while on high doses of corticosteroid therapy. Ophthalmology.

[90] Heeringa P, Rutgers A, Kallenberg CGM (2018). The net effect of ANCA on neutrophil extracellular trap formation. Kidney Int.

[91] Heineke MH, Ballering AV, Jamin A, Ben Mkaddem S, Monteiro RC, Van Egmond M (2017). New insights in the pathogenesis of immunoglobulin A vasculitis (Henoch-Schonlein purpura). Autoimmun Rev.

[92] Henderson HM (2009). Goodpasture's 1919 article on the etiology of influenza. Am J Med Sci.

[93] Hernandez-Rodriguez J, Alba MA, Prieto-Gonzalez S, Cid MC (2014). Diagnosis and classification of polyarteritis nodosa. J Autoimmun.

[94] Hoffman GS, Merkel PA, Brasington RD, Lenschow DJ, Liang P (2004). Anti-tumor necrosis factor therapy in patients with difficult to treat Takayasu arteritis. Arthritis Rheum.

[95] Horton BT, Magath TB, Brown GE (1932). An undescribed form of arteritis of temporal vessels. Mayo Clin Proc.

[96] Howard T, Ahmad K, Swanson JA, Misra S (2014). Polyarteritis nodosa. Tech Vasc Interv Radiol.

[97] Hughes LB, Bridges SL (2002). Polyarteritis nodosa and microscopic polyangiitis: etiologic and diagnostic considerations. Curr Rheumatol Rep.

[98] Hutchinson J (1890). On a peculiar form of thrombotic arteritis of the aged which is sometimes productive of gangrene. Arch Surg.

[99] Iannella G, Greco A, Granata G, Manno A, Pasquariello B, Angeletti D (2016). Granulomatosis with polyangiitis and facial palsy: Literature review and insight in the autoimmune pathogenesis. Autoimmun Rev.

[100] Iliescu DA, Timaru CM, Batras M, De Simone A, Stefan C (2015). Cogan's syndrome. Rom J Ophthalmol.

[101] International Study Group for Behcet's Disease (1990). Criteria for diagnosis of Behcet's disease. Lancet.

[102] Ishikawa K (1988). Diagnostic approach and proposed criteria for the clinical diagnosis of Takayasu's arteriopathy. J Am Coll Cardiol.

[103] Ishikawa K, Maetani S (1994). Long-term outcome for 120 Japanese patients with Takayasu's disease. Clinical and statistical analyses of related prognostic factors. Circulation.

[104] Jachiet M, Samson M, Cottin V, Kahn JE, Le Guenno G, Bonniaud P (2016). Anti-IgE monoclonal antibody (Omalizumab) in refractory and relapsing eosinophilic granulomatosis with polyangiitis (Churg-Strauss): data on seventeen patients. Arthritis Rheumatol.

[105] Jara LJ, Navarro C, Medina G, Vera-Lastra O, Saavedra MA (2009). Hypocomplementemic urticarial vasculitis syndrome. Curr Rheumatol Rep.

[106] Jennette JC, Falk RJ (1997). Small-vessel vasculitis. N Engl J Med.

[107] Jennette JC, Falk RJ, Andrassy K, Bacon PA, Churg J, Gross WL (1994). Nomenclature of systemic vasculitides. Proposal of an international consensus conference. Arthritis Rheum.

[108] Jennette JC, Falk RJ, Bacon PA, Basu N, Cid MC, Ferrario F (2013). 2012 revised International Chapel Hill Consensus Conference Nomenclature of Vasculitides. Arthritis Rheum.

[109] Jung DH, Nadol JB, Folkerth RD, Merola JF (2016). Histopathology of the inner ear in a case with recent onset of Cogan's syndrome: evidence for vasculitis. Ann Otol Rhinol Laryngol.

[110] Jung JH, Lee YH, Song GG, Jeong HS, Kim JH, Choi SJ (2018). Endovascular versus open surgical intervention in patients with Takayasu's arteritis: a meta-analysis. Eur J Vasc Endovasc Surg.

[111] Jung YS, Cheon JH, Hong SP, Kim TI, Kim WH (2012). Clinical outcomes and prognostic factors for thiopurine maintenance therapy in patients with intestinal Behcet's disease. Inflamm Bowel Dis.

[112] Jung YS, Hong SP, Kim TI, Kim WH, Cheon JH (2012). Long-term clinical outcomes and factors predictive of relapse after 5-aminosalicylate or sulfasalazine therapy in patients with intestinal Behcet disease. J Clin Gastroenterol.

[113] Kain R, Exner M, Brandes R, Ziebermayr R, Cunningham D, Alderson CA (2008). Molecular mimicry in pauci-immune focal necrotizing glomerulonephritis. Nat Med.

[114] Kallenberg CG (2007). Antineutrophil cytoplasmic autoantibody-associated small-vessel vasculitis. Curr Opin Rheumatol.

[115] Kaneko F, Oyama N, Yanagihori H, Isogai E, Yokota K, Oguma K (2008). The role of streptococcal hypersensitivity in the pathogenesis of Behcet's Disease. Eur J Dermatol.

[116] Kanzaki J (1994). Immune-mediated sensorineural hearing loss. Acta Otolaryngol Suppl.

[117] Katabathina VS, Katre R, Prasad SR, Surabhi VR, Shanbhogue AK, Sunnapwar A (2011). Wunderlich syndrome: cross-sectional imaging review. J Comput Assist Tomogr.

[118] Kawasaki T (1967). Arerugi.

[119] Kawasaki T (2006). Kawasaki disease. Proc Jpn Acad Ser B Phys Biol Sci.

[120] Keser G, Aksu K, Direskeneli H (2018). Takayasu arteritis: an update. Turk J Med Sci.

[121] Kessel A, Vadasz Z, Toubi E (2014). Cogan syndrome - pathogenesis, clinical variants and treatment approaches. Autoimmun Rev.

[122] Keystone EC (2004). The utility of tumour necrosis factor blockade in orphan diseases. Ann Rheum Dis.

[123] Kim ESH, Beckman J (2018). Takayasu arteritis: challenges in diagnosis and management. Heart.

[124] Kim H, Barra L (2018). Ischemic complications in Takayasu's arteritis: A meta-analysis. Semin Arthritis Rheum.

[125] Kim JJ, Yun SW, Yu JJ, Yoon KL, Lee KY, Kil HR (2017). A genome-wide association analysis identifies NMNAT2 and HCP5 as susceptibility loci for Kawasaki disease. J Hum Genet.

[126] King WJ, Schlieper A, Birdi N, Cappelli M, Korneluk Y, Rowe PC (2000). The effect of Kawasaki disease on cognition and behavior. Arch Pediatr Adolesc Med.

[127] Kiryluk K, Moldoveanu Z, Sanders JT, Eison TM, Suzuki H, Julian BA (2011). Aberrant glycosylation of IgA1 is inherited in both pediatric IgA nephropathy and Henoch-Schonlein purpura nephritis. Kidney Int.

[128] Kiykim AA, Horoz M, Gok E (2010). Successful treatment of resistant antiglomerular basement membrane antibody positivity with mycophenolic acid. Intern Med.

[129] Kobayashi S, Fujimoto S (2013). Epidemiology of vasculitides: differences between Japan, Europe and North America. Clin Exp Nephrol.

[130] Kobayashi S, Yano T, Matsumoto Y, Numano F, Nakajima N, Yasuda K (2003). Clinical and epidemiologic analysis of giant cell (temporal) arteritis from a nationwide survey in 1998 in Japan: the first government-supported nationwide survey. Arthritis Rheum.

[131] Kobayashi T, Inoue Y, Takeuchi K, Okada Y, Tamura K, Tomomasa T (2006). Prediction of intravenous immunoglobulin unresponsiveness in patients with Kawasaki disease. Circulation.

[132] Lamprecht P, Kerstein A, Klapa S, Schinke S, Karsten CM, Yu X (2018). Pathogenetic and clinical aspects of anti-neutrophil cytoplasmic autoantibody-associated vasculitides. Front Immunol.

[133] Langford CA, Cuthbertson D, Ytterberg SR, Khalidi N, Monach PA, Carette S (2017). A randomized, double-blind trial of Abatacept (CTLA-4Ig) for the treatment of Takayasu arteritis. Arthritis Rheumatol.

[134] Lau KK, Suzuki H, Novak J, Wyatt RJ (2010). Pathogenesis of Henoch-Schonlein purpura nephritis. Pediatr Nephrol.

[135] Laudien M, Gadola SD, Podschun R, Hedderich J, Paulsen J, Reinhold-Keller E (2010). Nasal carriage of Staphylococcus aureus and endonasal activity in Wegener s granulomatosis as compared to rheumatoid arthritis and chronic Rhinosinusitis with nasal polyps. Clin Exp Rheumatol.

[136] Lazarus B, John GT, O'Callaghan C, Ranganathan D (2016). Recent advances in anti-neutrophil cytoplasmic antibody-associated vasculitis. Indian J Nephrol.

[137] Lee JJY, Alsaleem A, Chiang GPK, Limenis E, Sontichai W, Yeung RSM (2019). Hallmark trials in ANCA-associated vasculitis (AAV) for the pediatric rheumatologist. Pediatr Rheumatol Online J.

[138] Lee S, Bang D, Cho YH, Lee ES, Sohn S (1996). Polymerase chain reaction reveals herpes simplex virus DNA in saliva of patients with Behcet's disease. Arch Dermatol Res.

[139] Lerner AB, Watson CJ (1947). Studies of cryoglobulins;unusual purpura associated with the presence of a high concentration of cryoglobulin (cold precipitable serum globulin). Am J Med Sci.

[140] Levy JB, Turner AN, Rees AJ, Pusey CD (2001). Long-term outcome of anti-glomerular basement membrane antibody disease treated with plasma exchange and immunosuppression. Ann Intern Med.

[141] Lew W, Chang JY, Jung JY, Bang D (2008). Increased expression of interleukin-23 p19 mRNA in erythema nodosum-like lesions of Behcet's disease. Br J Dermatol.

[142] Lhote F, Guillevin L (1995). Polyarteritis nodosa, microscopic polyangiitis, and Churg-Strauss syndrome. Clinical aspects and treatment. Rheum Dis Clin North Am.

[143] Licciardi F, Pruccoli G, Denina M, Parodi E, Taglietto M, Rosati S (2020). SARS-CoV-2-induced Kawasaki-like hyperinflammatory syndrome: a novel covid phenotype in children. Pediatrics.

[144] Lie JT (1989). Systemic and isolated vasculitis. A rational approach to classification and pathologic diagnosis. Pathol Annu.

[145] Lightfoot RW, Michel BA, Bloch DA, Hunder GG, Zvaifler NJ, McShane DJ (1990). The American College of Rheumatology 1990 criteria for the classification of polyarteritis nodosa. Arthritis Rheum.

[146] Lilliebladh S, Johansson A, Pettersson A, Ohlsson S, Hellmark T (2018). Phenotypic characterization of circulating CD4(+) T cells in ANCA-associated vasculitis. J Immunol Res.

[147] Liu GT, Glaser JS, Schatz NJ, Smith JL (1994). Visual morbidity in giant cell arteritis. Clinical characteristics and prognosis for vision. Ophthalmology.

[148] Lopez-Mejias R, Carmona FD, Castaneda S, Genre F, Remuzgo-Martinez S, Sevilla-Perez B (2017). A genome-wide association study suggests the HLA Class II region as the major susceptibility locus for IgA vasculitis. Sci Rep.

[149] Lopez-Mejias R, Castaneda S, Genre F, Remuzgo-Martinez S, Carmona FD, Llorca J (2018). Genetics of immunoglobulin-A vasculitis (Henoch-Schonlein purpura): An updated review. Autoimmun Rev.

[150] Lopez-Mejias R, Genre F, Perez BS, Castaneda S, Ortego-Centeno N, Llorca J (201). Association of HLA-B*41:02 with Henoch-Schonlein Purpura (IgA Vasculitis) in Spanish individuals irrespective of the HLA-DRB1 status. Arthritis Res Ther.

[151] Lopez-Mejias R, Genre F, Perez BS, Castaneda S, Ortego-Centeno N, Llorca J (2015). HLA-DRB1 association with Henoch-Schonlein purpura. Arthritis Rheumatol.

[152] Lunardi C, Bason C, Leandri M, Navone R, Lestani M, Millo E (2002). Autoantibodies to inner ear and endothelial antigens in Cogan's syndrome. Lancet.

[153] Lyons PA, Peters JE, Alberici F, Liley J, Coulson RMR, Astle W (2019). Genome-wide association study of eosinophilic granulomatosis with polyangiitis reveals genomic loci stratified by ANCA status. Nat Commun.

[154] Mahr A, Guillevin L, Poissonnet M, Ayme S (2004). Prevalences of polyarteritis nodosa, microscopic polyangiitis, Wegener's granulomatosis, and Churg-Strauss syndrome in a French urban multiethnic population in 2000: a capture-recapture estimate. Arthritis Rheum.

[155] Makino N, Nakamura Y, Yashiro M, Ae R, Tsuboi S, Aoyama Y (2015). Descriptive epidemiology of Kawasaki disease in Japan, 2011-2012: from the results of the 22nd nationwide survey. J Epidemiol.

[156] Maldini C, Druce K, Basu N, LaValley MP, Mahr A (2018). Exploring the variability in Behcet's disease prevalence: a meta-analytical approach. Rheumatology (Oxford).

[157] Marder RJ, Burch FX, Schmid FR, Zeiss CR, Gewurz H (1978). Low molecular weight C1q-precipitins in hypocomplementemic vasculitis-urticaria syndrome: partial purification and characterization as immunoglobulin. J Immunol.

[158] Mason JC (2018). Surgical intervention and its role in Takayasu arteritis. Best Pract Res Clin Rheumatol.

[159] Mason JC (2015). Takayasu arteritis: surgical interventions. Curr Opin Rheumatol.

[160] Mason JC (2010). Takayasu arteritis--advances in diagnosis and management. Nat Rev Rheumatol.

[161] McAdoo SP, Pusey CD (2017). Anti-glomerular basement membrane disease. Clin J Am Soc Nephrol.

[162] McCrindle BW, Rowley AH, Newburger JW, Burns JC, Bolger AF, Gewitz M (2017). Diagnosis, treatment, and long-term management of Kawasaki disease: a scientific statement for health professionals from the American Heart Association. Circulation.

[163] Mehregan DR, Hall MJ, Gibson LE (1992). Urticarial vasculitis: a histopathologic and clinical review of 72 cases. J Am Acad Dermatol.

[164] Mekinian A, Resche-Rigon M, Comarmond C, Soriano A, Constans J, Alric L (2018). Efficacy of tocilizumab in Takayasu arteritis: Multicenter retrospective study of 46 patients. J Autoimmun.

[165] Mendes D, Correia M, Barbedo M, Vaio T, Mota M, Goncalves O (2009). Behcet's disease--a contemporary review. J Autoimmun.

[166] Miloslavsky EM, Niles JL, Wallace ZS, Cortazar FB, Fernandes A, Laliberte K (2018). Reducing glucocorticoid duration in ANCA-associated vasculitis: A pilot trial. Semin Arthritis Rheum.

[167] Mohammad AJ, Nilsson JA, Jacobsson LT, Merkel PA, Turesson C (2015). Incidence and mortality rates of biopsy-proven giant cell arteritis in southern Sweden. Ann Rheum Dis.

[168] Monach PA (2014). Biomarkers in vasculitis. Curr Opin Rheumatol.

[169] Mori M, Nwaogwugwu U, Akers GR, McGill RL (2013). Anti-glomerular basement membrane disease treated with mycophenolate mofetil, corticosteroids, and plasmapheresis. Clin Nephrol.

[170] Morton LT, Situnayake D, Wallace GR (2016). Genetics of Behcet's disease. Curr Opin Rheumatol.

[171] Muchtar E, Magen H, Gertz MA (2017). How I treat cryoglobulinemia. Blood.

[172] Murchison AP, Bilyk JR, Eagle RC, Savino PJ (2012). Shrinkage revisited: how long is long enough?. Ophthalmic Plast Reconstr Surg.

[173] Nakamura Y (2018). Kawasaki disease: epidemiology and the lessons from it. Int J Rheum Dis.

[174] Nakamura Y, Yashiro M, Yamashita M, Aoyama N, Otaki U, Ozeki Y (2018). Cumulative incidence of Kawasaki disease in Japan. Pediatr Int.

[175] Nakaoka Y, Isobe M, Takei S, Tanaka Y, Ishii T, Yokota S (2018). Efficacy and safety of tocilizumab in patients with refractory Takayasu arteritis: results from a randomised, double-blind, placebo-controlled, phase 3 trial in Japan (the TAKT study). Ann Rheum Dis.

[176] Netzer KO, Leinonen A, Boutaud A, Borza DB, Todd P, Gunwar S (1999). The goodpasture autoantigen. Mapping the major conformational epitope(s) of alpha3(IV) collagen to residues 17-31 and 127-141 of the NC1 domain. J Biol Chem.

[177] Newburger JW, Takahashi M, Beiser AS, Burns JC, Bastian J, Chung KJ (1991). A single intravenous infusion of gamma globulin as compared with four infusions in the treatment of acute Kawasaki syndrome. N Engl J Med.

[178] Niederkohr RD, Levin LA (2005). Management of the patient with suspected temporal arteritis a decision-analytic approach. Ophthalmology.

[179] Nong BR, Huang YF, Chuang CM, Liu CC, Hsieh KS (2007). Fifteen-year experience of children with Henoch-Schonlein purpura in southern Taiwan, 1991-2005. J Microbiol Immunol Infect.

[180] Oni L, Sampath S (2019). Childhood IgA vasculitis (Henoch Schonlein Purpura)-advances and knowledge gaps. Front Pediatr.

[181] Onouchi Y (2018). The genetics of Kawasaki disease. Int J Rheum Dis.

[182] Orsoni JG, Lagana B, Rubino P, Zavota L, Bacciu S, Mora P (2010). Rituximab ameliorated severe hearing loss in Cogan's syndrome: a case report. Orphanet J Rare Dis.

[183] Ozdemir M, Balevi S, Deniz F, Mevlitoglu I (2007). Pathergy reaction in different body areas in Behcet's disease. Clin Exp Dermatol.

[184] Ozen S, Pistorio A, Iusan SM, Bakkaloglu A, Herlin T, Brik R (2010). EULAR/PRINTO/PRES criteria for Henoch-Schonlein purpura, childhood polyarteritis nodosa, childhood Wegener granulomatosis and childhood Takayasu arteritis: Ankara 2008. Part II: Final classification criteria. Ann Rheum Dis.

[185] Ozen S, Ruperto N, Dillon MJ, Bagga A, Barron K, Davin JC (2006). EULAR/PReS endorsed consensus criteria for the classification of childhood vasculitides. Ann Rheum Dis.

[186] Pagnoux C, Seror R, Henegar C, Mahr A, Cohen P, Le Guern V (2010). Clinical features and outcomes in 348 patients with polyarteritis nodosa: a systematic retrospective study of patients diagnosed between 1963 and 2005 and entered into the French Vasculitis Study Group Database. Arthritis Rheum.

[187] Park MC, Lee SW, Park YB, Chung NS, Lee SK (2005). Clinical characteristics and outcomes of Takayasu's arteritis: analysis of 108 patients using standardized criteria for diagnosis, activity assessment, and angiographic classification. Scand J Rheumatol.

[188] Parthasarathy P, Agarwal A, Chawla K, Tofighi T, Mondal TK (2015). Upcoming biomarkers for the diagnosis of Kawasaki disease: A review. Clin Biochem.

[189] Pileri P, Uematsu Y, Campagnoli S, Galli G, Falugi F, Petracca R (1998). Binding of hepatitis C virus to CD81. Science.

[190] Piram M, Maldini C, Biscardi S, De Suremain N, Orzechowski C, Georget E (2017). Incidence of IgA vasculitis in children estimated by four-source capture-recapture analysis: a population-based study. Rheumatology (Oxford).

[191] Ralli M, D'Aguanno V, Di Stadio A, De Virgilio A, Croce A, Longo L (2018a). Audiovestibular symptoms in systemic autoimmune diseases. J Immunol Res.

[192] Ralli M, Di Stadio A, De Virgilio A, Croce A, de Vincentiis M (2018b). Autoimmunity and otolaryngology diseases. J Immunol Res.

[193] Ralli M, Greco A, de Vincentiis M (2017). Hearing loss in Takayasu's arteritis: a role for hyperbaric oxygen therapy?. J Int Adv Otol.

[194] Ralli M, Greco A, Falasca V, Altissimi G, Tombolini M, Turchetta R (2017). Recovery from repeated sudden hearing loss in a patient with Takayasu's arteritis treated with hyperbaric oxygen therapy: the first report in the literature. Case Rep Otolaryngol.

[195] Ramos-Casals M, Trejo O, Garcia-Carrasco M, Cervera R, Font J (2000). Mixed cryoglobulinemia: new concepts. Lupus.

[196] Reinhold-Keller E, Herlyn K, Wagner-Bastmeyer R, Gross WL (2005). Stable incidence of primary systemic vasculitides over five years: results from the German vasculitis register. Arthritis Rheum.

[197] Renauer P, Sawalha AH (2017). The genetics of Takayasu arteritis. Presse Med.

[198] Renauer PA, Saruhan-Direskeneli G, Coit P, Adler A, Aksu K, Keser G (2015). Identification of susceptibility loci in IL6, RPS9/LILRB3, and an intergenic locus on chromosome 21q22 in Takayasu arteritis in a genome-wide association study. Arthritis Rheumatol.

[199] Robson J, Doll H, Suppiah R, Flossmann O, Harper L, Hoglund P (2015). Damage in the anca-associated vasculitides: long-term data from the European vasculitis study group (EUVAS) therapeutic trials. Ann Rheum Dis.

[200] Roccatello D, Fornasieri A, Giachino O, Rossi D, Beltrame A, Banfi G (2007). Multicenter study on hepatitis C virus-related cryoglobulinemic glomerulonephritis. Am J Kidney Dis.

[201] Roccatello D, Saadoun D, Ramos-Casals M, Tzioufas AG, Fervenza FC, Cacoub P (2018). Cryoglobulinaemia. Nat Rev Dis Primers.

[202] Rovin BH, Caster DJ, Cattran DC, Gibson KL, Hogan JJ, Moeller MJ (2019). Management and treatment of glomerular diseases (part 2): conclusions from a Kidney Disease: Improving Global Outcomes (KDIGO) Controversies Conference. Kidney Int.

[203] Rypdal M, Rypdal V, Burney JA, Cayan D, Bainto E, Skochko S (2018). Clustering and climate associations of Kawasaki Disease in San Diego County suggest environmental triggers. Sci Rep.

[204] Saadoun D, Asli B, Wechsler B, Houman H, Geri G, Desseaux K (2012). Long-term outcome of arterial lesions in Behcet disease: a series of 101 patients. Medicine (Baltimore).

[205] Sag E, Batu ED, Ozen S (2017). Childhood systemic vasculitis. Best Pract Res Clin Rheumatol.

[206] Saigal K, Valencia IC, Cohen J, Kerdel FA (2003). Hypocomplementemic urticarial vasculitis with angioedema, a rare presentation of systemic lupus erythematosus: rapid response to rituximab. J Am Acad Dermatol.

[207] Sakane T, Takeno M, Suzuki N, Inaba G (1999). Behcet's disease. N Engl J Med.

[208] Salmela A, Rasmussen N, Tervaert JWC, Jayne DRW, Ekstrand A, European Vasculitis Study Group (2017). Chronic nasal Staphylococcus aureus carriage identifies a subset of newly diagnosed granulomatosis with polyangiitis patients with high relapse rate. Rheumatology (Oxford).

[209] Salvarani C, Cantini F, Hunder GG (2008). Polymyalgia rheumatica and giant-cell arteritis. Lancet.

[210] Sangolli PM, Lakshmi DV (2019). Vasculitis: A checklist to approach and treatment update for dermatologists. Indian Dermatol Online J.

[211] Sardu C, Gambardella J, Morelli MB, Wang X, Marfella R, Santulli G (2020). Hypertension, thrombosis, kidney failure, and diabetes: Is COVID-19 an endothelial disease? A comprehensive evaluation of clinical and basic evidence. J Clin Med.

[212] Sargur R, White P, Egner W (2010). Cryoglobulin evaluation: best practice?. Ann Clin Biochem.

[213] Saus J, Wieslander J, Langeveld JP, Quinones S, Hudson BG (1988). Identification of the Goodpasture antigen as the alpha 3(IV) chain of collagen IV. J Biol Chem.

[214] Schmidt WA (2013). Imaging in vasculitis. Best Pract Res Clin Rheumatol.

[215] Schmitt WH, van der Woude FJ (2004). Clinical applications of antineutrophil cytoplasmic antibody testing. Curr Opin Rheumatol.

[216] Schnabel A, Hedrich CM (2018). Childhood vasculitis. Front Pediatr.

[217] Schwartz HR, McDuffie FC, Black LF, Schroeter AL, Conn DL (1982). Hypocomplementemic urticarial vasculitis: association with chronic obstructive pulmonary disease. Mayo Clin Proc.

[218] Seyahi E (2017). Takayasu arteritis: an update. Curr Opin Rheumatol.

[219] Shah MK, Hugghins SY (2002). Characteristics and outcomes of patients with Goodpasture's syndrome. South Med J.

[220] Shim J, Lee ES, Park S, Bang D, Sohn S (2011). CD4(+) CD25(+) regulatory T cells ameliorate Behcet's disease-like symptoms in a mouse model. Cytotherapy.

[221] Sidana S, Rajkumar SV, Dispenzieri A, Lacy MQ, Gertz MA, Buadi FK (2017). Clinical presentation and outcomes of patients with type 1 monoclonal cryoglobulinemia. Am J Hematol.

[222] Siegert C, Daha M, Westedt ML, van der Voort E, Breedveld F (1991). IgG autoantibodies against C1q are correlated with nephritis, hypocomplementemia, and dsDNA antibodies in systemic lupus erythematosus. J Rheumatol.

[223] Silva F, Pinto C, Barbosa A, Borges T, Dias C, Almeida J (2019). New insights in cryoglobulinemic vasculitis. J Autoimmun.

[224] Simpfendorfer KR, Armstead BE, Shih A, Li W, Curran M, Manjarrez-Orduno N (2015). Autoimmune disease-associated haplotypes of BLK exhibit lowered thresholds for B cell activation and expansion of Ig class-switched B cells. Arthritis Rheumatol.

[225] Sjowall C, Mandl T, Skattum L, Olsson M, Mohammad AJ (2018). Epidemiology of hypocomplementaemic urticarial vasculitis (anti-C1q vasculitis). Rheumatology (Oxford).

[226] Soto ME, Montufar-Robles I, Jimenez-Morales S, Gamboa R, Huesca-Gomez C, Ramirez-Bello J (2019). An association study in PTPN22 suggests that is a risk factor to Takayasu's arteritis. Inflamm Res.

[227] Tacke CE, Haverman L, Berk BM, van Rossum MA, Kuipers IM, Grootenhuis MA (2012). Quality of life and behavioral functioning in Dutch children with a history of Kawasaki disease. J Pediatr.

[228] Takada S, Shimizu T, Hadano Y, Matsumoto K, Kataoka Y, Arima Y (2012). Cryoglobulinemia (review). Mol Med Rep.

[229] Terai M, Shulman ST (1997). Prevalence of coronary artery abnormalities in Kawasaki disease is highly dependent on gamma globulin dose but independent of salicylate dose. J Pediatr.

[230] Terao C (2016). Revisited HLA and non-HLA genetics of Takayasu arteritis - where are we? J Hum Genet.

[231] Terao C, Yoshifuji H, Matsumura T, Naruse TK, Ishii T, Nakaoka Y (2018). Genetic determinants and an epistasis of LILRA3 and HLA-B*52 in Takayasu arteritis. Proc Natl Acad Sci U S A.

[232] Terao C, Yoshifuji H, Nakajima T, Yukawa N, Matsuda F, Mimori T (2016). Ustekinumab as a therapeutic option for Takayasu arteritis: from genetic findings to clinical application. Scand J Rheumatol.

[233] Terrier B, Karras A, Kahn JE, Le Guenno G, Marie I, Benarous L (2013). The spectrum of type I cryoglobulinemia vasculitis: new insights based on 64 cases. Medicine (Baltimore).

[234] Tesar V, Kazderova M, Hlavackova L (2004). Rokitansky and his first description of polyarteritis nodosa. J Nephrol.

[235] Tombetti E, Mason JC (2019). Takayasu arteritis: advanced understanding is leading to new horizons. Rheumatology (Oxford).

[236] Toshihiko N (1996). Current status of large and small vessel vasculitis in Japan. Int J Cardiol.

[237] Touzot M, Poisson J, Faguer S, Ribes D, Cohen P, Geffray L (2015). Rituximab in anti-GBM disease: A retrospective study of 8 patients. J Autoimmun.

[238] Tracy A, Subramanian A, Adderley NJ, Cockwell P, Ferro C, Ball S (2019). Cardiovascular, thromboembolic and renal outcomes in IgA vasculitis (Henoch-Schonlein purpura): a retrospective cohort study using routinely collected primary care data. Ann Rheum Dis.

[239] Trapani S, Micheli A, Grisolia F, Resti M, Chiappini E, Falcini F (2005). Henoch Schonlein purpura in childhood: epidemiological and clinical analysis of 150 cases over a 5-year period and review of literature. Semin Arthritis Rheum.

[240] Trejo O, Ramos-Casals M, Garcia-Carrasco M, Yague J, Jimenez S, de la Red G (2001). Cryoglobulinemia: study of etiologic factors and clinical and immunologic features in 443 patients from a single center. Medicine (Baltimore).

[241] Trepo C, Guillevin L (2001). Polyarteritis nodosa and extrahepatic manifestations of HBV infection: the case against autoimmune intervention in pathogenesis. J Autoimmun.

[242] Turner N, Mason PJ, Brown R, Fox M, Povey S, Rees A (1992). Molecular cloning of the human Goodpasture antigen demonstrates it to be the alpha 3 chain of type IV collagen. J Clin Invest.

[243] Uehara R, Yashiro M, Nakamura Y, Yanagawa H (2004). Clinical features of patients with Kawasaki disease whose parents had the same disease. Arch Pediatr Adolesc Med.

[244] van Oers HA, Tacke CE, Haverman L, Kuipers IM, Maurice-Stam H, Kuijpers TW (2014). Health related quality of life and perceptions of child vulnerability among parents of children with a history of Kawasaki disease. Acta Paediatr.

[245] Vanoli M, Daina E, Salvarani C, Sabbadini MG, Rossi C, Bacchiani G (2005). Takayasu's arteritis: A study of 104 Italian patients. Arthritis Rheum.

[246] Verity DH, Marr JE, Ohno S, Wallace GR, Stanford MR (1999). Behcet's disease, the Silk Road and HLA-B51: historical and geographical perspectives. Tissue Antigens.

[247] Vollertsen RS, McDonald TJ, Younge BR, Banks PM, Stanson AW, Ilstrup DM (1986). Cogan's syndrome: 18 cases and a review of the literature. Mayo Clin Proc.

[248] Wallace GR (2014). HLA-B*51 the primary risk in Behcet disease. Proc Natl Acad Sci U S A.

[249] Watts RA, Mahr A, Mohammad AJ, Gatenby P, Basu N, Flores-Suarez LF (2015). Classification, epidemiology and clinical subgrouping of antineutrophil cytoplasmic antibody (ANCA)-associated vasculitis. Nephrol Dial Transplant.

[250] Watts RA, Robson J (2018). Introduction, epidemiology and classification of vasculitis. Best Pract Res Clin Rheumatol.

[251] Wendt M, Borjesson O, Avik A, Bratt J, Anderstam B, Qureshi AR (2013). Macrophage migration inhibitory factor (MIF) and thyroid hormone alterations in antineutrophil cytoplasmic antibody (ANCA)-associated vasculitis (AAV). Mol Med.

[252] Westman KW, Selga D, Isberg PE, Bladstrom A, Olsson H (2003). High proteinase 3-anti-neutrophil cytoplasmic antibody (ANCA) level measured by the capture enzyme-linked immunosorbent assay method is associated with decreased patient survival in ANCA-associated vasculitis with renal involvement. J Am Soc Nephrol.

[253] Wisnieski JJ, Baer AN, Christensen J, Cupps TR, Flagg DN, Jones JV (1995). Hypocomplementemic urticarial vasculitis syndrome. Clinical and serologic findings in 18 patients. Medicine (Baltimore).

[254] Wisnieski JJ, Jones SM (1992). IgG autoantibody to the collagen-like region of Clq in hypocomplementemic urticarial vasculitis syndrome, systemic lupus erythematosus, and 6 other musculoskeletal or rheumatic diseases. J Rheumatol.

[255] Wulffraat NM, Vastert B, SHARE consortium (2013). Time to share. Pediatr Rheumatol Online J.

[256] Yates M, MacGregor AJ, Robson J, Craven A, Merkel PA, Luqmani RA (2017). The association of vascular risk factors with visual loss in giant cell arteritis. Rheumatology (Oxford).

[257] Yates M, Watts RA, Bajema IM, Cid MC, Crestani B, Hauser T (2016). EULAR/ERA-EDTA recommendations for the management of ANCA-associated vasculitis. Ann Rheum Dis.

[258] Younger DS (2019). Giant cell arteritis. Neurol Clin.

[259] Youngstein T, Peters JE, Hamdulay SS, Mewar D, Price-Forbes A, Lloyd M (2014). Serial analysis of clinical and imaging indices reveals prolonged efficacy of TNF-alpha and IL-6 receptor targeted therapies in refractory Takayasu arteritis. Clin Exp Rheumatol.

[260] Yurdakul S, Mat C, Tuzun Y, Ozyazgan Y, Hamuryudan V, Uysal O (2001). A double-blind trial of colchicine in Behcet's syndrome. Arthritis Rheum.

[261] Zhang H, Watanabe R, Berry GJ, Vaglio A, Liao YJ, Warrington KJ (2017). Immunoinhibitory checkpoint deficiency in medium and large vessel vasculitis. Proc Natl Acad Sci U S A.

[262] Zhang YY, Tang Z, Chen DM, Gong DH, Ji DX, Liu ZH (2014). Comparison of double filtration plasmapheresis with immunoadsorption therapy in patients with anti-glomerular basement membrane nephritis. BMC Nephrol.

[263] Zouboulis CC (1999). Epidemiology of Adamantiades-Behcet's disease. Ann Med Interne (Paris).

